# The tumor mutational landscape of *BRCA2*-deficient primary and metastatic prostate cancer

**DOI:** 10.1038/s41698-022-00284-6

**Published:** 2022-06-17

**Authors:** Kevin H. Kensler, Shakuntala Baichoo, Shailja Pathania, Timothy R. Rebbeck

**Affiliations:** 1 Department of Population Health Sciences, Weill Cornell Medicine, New York, NY USA; 2 Department of Digital Technologies, FoICDT, University of Mauritius, Réduit, Mauritius; 3 Center for Personalized Cancer Therapy, University of Massachusetts, Boston, MA USA; 4 Department of Biology, University of Massachusetts, Boston, MA USA; 5 Division of Population Sciences, Dana-Farber Cancer Institute, Boston, MA USA; 6 Department of Epidemiology, Harvard T.H. Chan School of Public Health, Boston, MA USA

**Keywords:** Cancer genomics, Urological cancer

## Abstract

Carriers of germline *BRCA2* pathogenic sequence variants have elevated aggressive prostate cancer risk and are candidates for precision oncology treatments. We examined whether *BRCA2*-deficient (*BRCA2*^*d*^) prostate tumors have distinct genomic alterations compared with *BRCA2*-intact (*BRCA2*^*i*^) tumors. Among 2536 primary and 899 metastatic prostate tumors from the ICGC, GENIE, and TCGA databases, we identified 138 primary and 85 metastatic *BRCA2*^*d*^ tumors. Total tumor mutation burden (TMB) was higher among primary *BRCA2*^*d*^ tumors, although pathogenic TMB did not differ by tumor *BRCA2* status. Pathogenic and total single nucleotide variant (SNV) frequencies at *KMT2D* were higher in *BRCA2*^*d*^ primary tumors, as was the total SNV frequency at *KMT2D* in *BRCA2*^*d*^ metastatic tumors. Homozygous deletions at *NEK3*, *RB1*, and *APC* were enriched in *BRCA2*^*d*^ primary tumors, and *RB1* deletions in metastatic *BRCA2*^*d*^ tumors as well. *TMPRSS2*-*ETV1* fusions were more common in *BRCA2*^*d*^ tumors. These results identify somatic alterations that hallmark etiological and prognostic differences between *BRCA2*^*d*^ and *BRCA2*^*i*^ prostate tumors.

## Introduction

Male carriers of *BRCA2* germline pathogenic sequence variants (PSV) experience 2.6-fold higher lifetime risk of prostate cancer and a 7.3–8.6-fold higher risk of developing early-onset (<65 years) prostate cancer^1–3^. Germline *BRCA2* PSV are associated with higher tumor stage, Gleason grade, and prostate-specific antigen (PSA) levels at diagnosis^4–6^. Intraductal carcinoma of the prostate (IDCP) occurs more commonly in tumors that harbor a germline *BRCA2* mutation than in sporadic prostate cancers, and likewise confers a higher risk of mortality^7^. Prostate cancers with a germline *BRCA2* PSV are associated with higher rates of lymph node involvement, metastases, and prostate cancer-specific death for both primary and metastatic cancers^4,8,9^. Germline or somatic *BRCA2* loss occurs in ~13% of metastatic prostate cancers, compared with 3% in primary tumors^10,11^. The presence of a germline *BRCA2* PSV also directs therapeutic management with PARP inhibitors, although PARP inhibitors are not yet uniformly available globally^12^.

Prostate tumors that are *BRCA2*-deficient (*BRCA2*^*d*^) have aggressive genomic profiles that may contribute to the worse outcomes observed in this subset. Compared with *BRCA2*-intact (*BRCA2*^*i*^) tumors that do not contain a PSV, germline *BRCA2*^*d*^ prostate tumors have an elevated proportion of the genome altered^7^. The mean count of copy number alterations in prostate cancers was reported to be 3-fold higher among germline carriers of *BRCA2* PSV relative to non-carriers, with gains considerably more common in the region encompassing *c-MYC*^13^. Amplifications of the Wnt/β-catenin pathway modulators *MED12* and *MED12L* were also more common among germline *BRCA2*^*d*^ tumors^7^. Additionally, germline *BRCA2*^*d*^ prostate tumors have been shown to experience global hypomethylation relative to *BRCA2*^*i*^ tumors^7^.

We hypothesize that *BRCA2*^d^ tumors represent a unique phenotype in prostate cancer. Identification of tumor genomic aberrations in *BRCA2*^*d*^ prostate tumors may provide insight into the mechanisms of *BRCA2*-associated prostate carcinogenesis and progression, which could have downstream implications for prevention or therapeutics. We assembled multi-omic data including single nucleotide variants (SNVs), copy number alterations (CNAs), and structural variants (SVs) from multiple public databases to create the largest and most comprehensive dataset of *BRCA2*^*d*^ prostate tumors to date and compared these with *BRCA2*^*i*^ prostate tumors.

## Results

### BRCA2^d^ prostate tumors

**Fig. 1:**
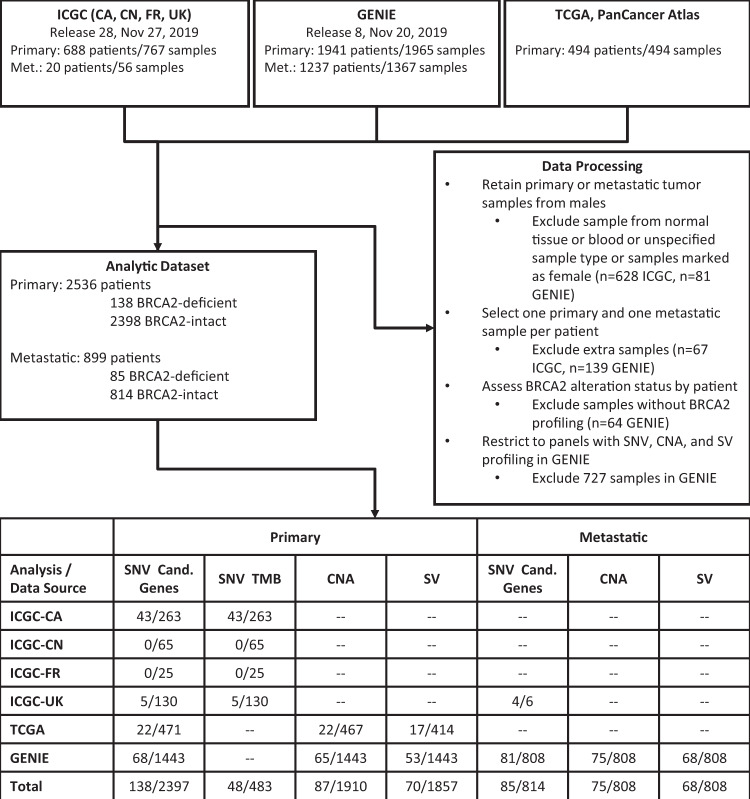
Diagram of workflow and data processing steps to generate analytic datasets. Bottom table displays number of samples (*BRCA2*^*d*^ samples/ *BRCA2*^*i*^ samples) included in each analysis. *BRCA2*^*d*^ *BRCA2*-deficient; *BRCA2*^*i*^ *BRCA2*-intact; Cand. Gens Candidate genes.

A total of 2536 primary and 899 metastatic prostatic adenocarcinomas from the International Cancer Genome Consortium (ICGC)^14^, the American Association for Cancer Research Project Genomics Evidence Neoplasia Information Exchange (GENIE)^15^, and The Cancer Genome Atlas (TCGA) databases^16^ were identified with available SNV, CNA, or SV) data (Fig. 1). One hundred thirty-eight primary tumors (5.4%) harbored a somatic PSV leading to *BRCA2*^*d*^, while 2398 tumors (94.6%) had no alterations or a non-pathogenic alteration at *BRCA2* and were denoted *BRCA2*^*i*^. Fifty-three primary *BRCA2*^*d*^ tumors had pathogenic SNVs, 74 had CNAs, and seven had SVs affecting *BRCA2*, while the remaining four tumors had multiple PSV affecting *BRCA2* (Supplementary Table 1). Patients with losses of heterozygosity (LOH; *n* = 184) or non-pathogenic SNVs (*n* = 68) at BRCA2 were considered *BRCA2*^*i*^.

Eighty-five metastatic tumors (9.5%) were adjudicated to be *BRCA2*^*d*^, while 814 (90.5%) were *BRCA2*^*i*^. Among the metastatic *BRCA2*^*d*^ tumors, 36 harbored pathogenic SNVs, 39 had CNAs, and five had SVs affecting *BRCA2*, and the remaining five tumors had multiple PSVs at *BRCA2* (Supplementary Table 1). There were 40 *BRCA2*^*i*^ patients with LOH at BRCA2 and 22 patients with non-pathogenic SNVs. Clinical and pathological characteristics of primary and metastatic tumors by tumor *BRCA2* status are shown in Supplementary Table 3.

### Single nucleotide variant (SNV) analyses

**Fig. 2:**
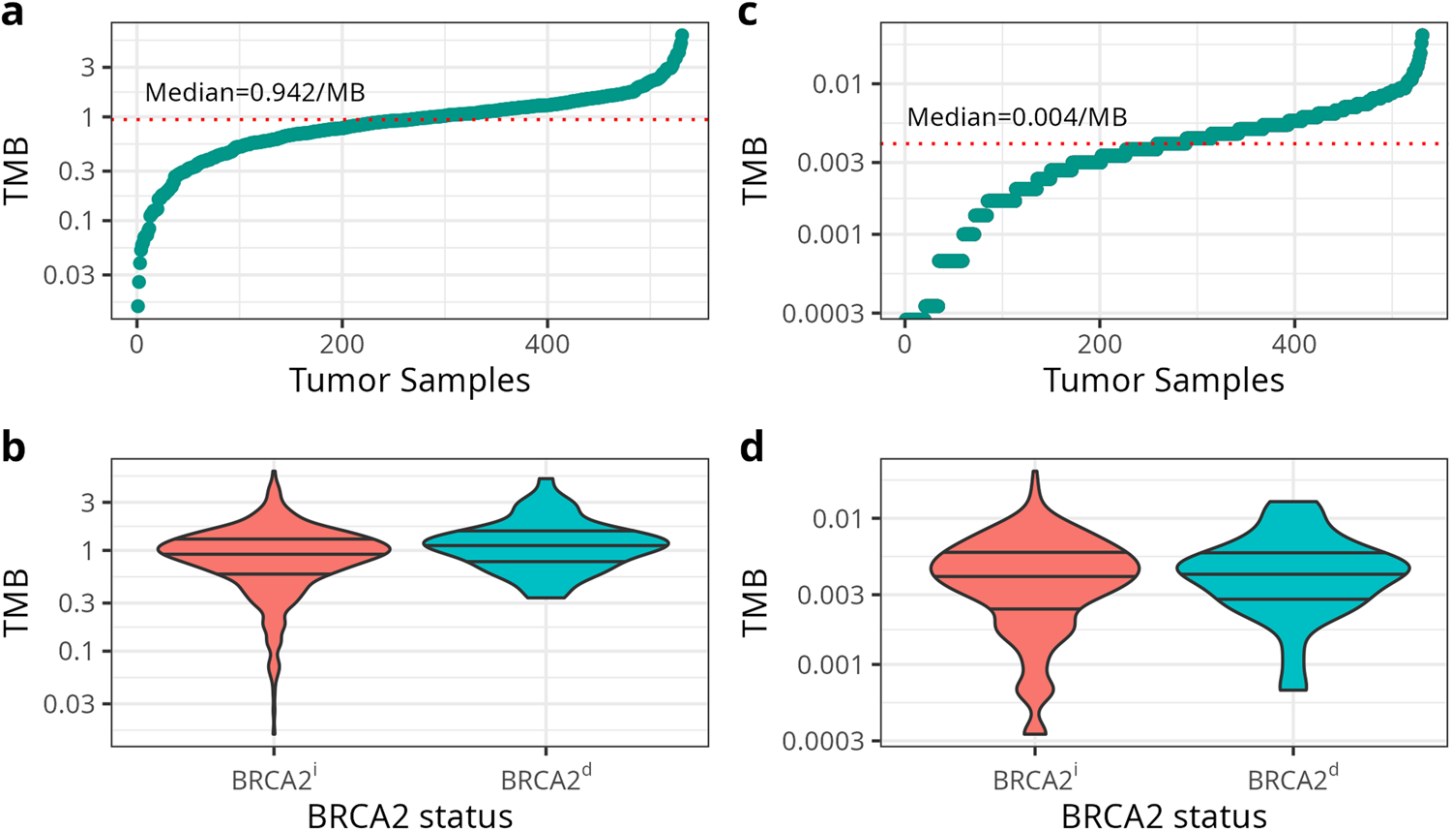
Tumor Mutational Burden (TMB) (mutations per megabase) in primary *BRCA2*-deficient and *BRCA2*-intact tumor samples. Panels display **a** the distribution of total TMB in all tumors, **b** the distribution of total TMB by *BRCA2* status, **c** the distribution of pathogenic TMB in all tumors, and **d** the distribution of pathogenic TMB by *BRCA2* status. TMB is estimated from samples with whole genome sequencing from the ICGC dataset (*n* = 531). In panels b and d, the lower, middle, and upper lines correspond to the lower quartile, median, and upper quartile of TMB, respectively. *P*-values are from a two-sided Wilcoxon rank-sum test. *BRCA2*^*d*^ *BRCA2*-deficient; *BRCA2*^*i*^ *BRCA2*-intact.

Among 531 patients with primary tumors from the ICGC database, the median SNV TMB was 0.942 per mb (range: 0.015–6.111) (Fig. 2a). The median SNV TMB in *BRCA2*^*d*^ tumors (median = 1.103, range: 0.338–5.152) was significantly higher than among *BRCA2*^*i*^ tumors (median = 0.925, range: 0.015–6.111; *p* = 0.011 from Wilcoxon rank-sum test) (Fig. 2b). The median pathogenic SNV TMB was 0.004/mb (range: 0.000–0.021) (Fig. 2c). However, in contrast to the total SNV TMB, there was no difference in the pathogenic SNV TMB between *BRCA2*^*d*^ tumors (median = 0.004, range: 0.000–0.013) and *BRCA2*^*i*^ tumors (median = 0.004, range: 0.000–0.021; *p* = 0.20) (Fig. 2d).

**Fig. 3:**
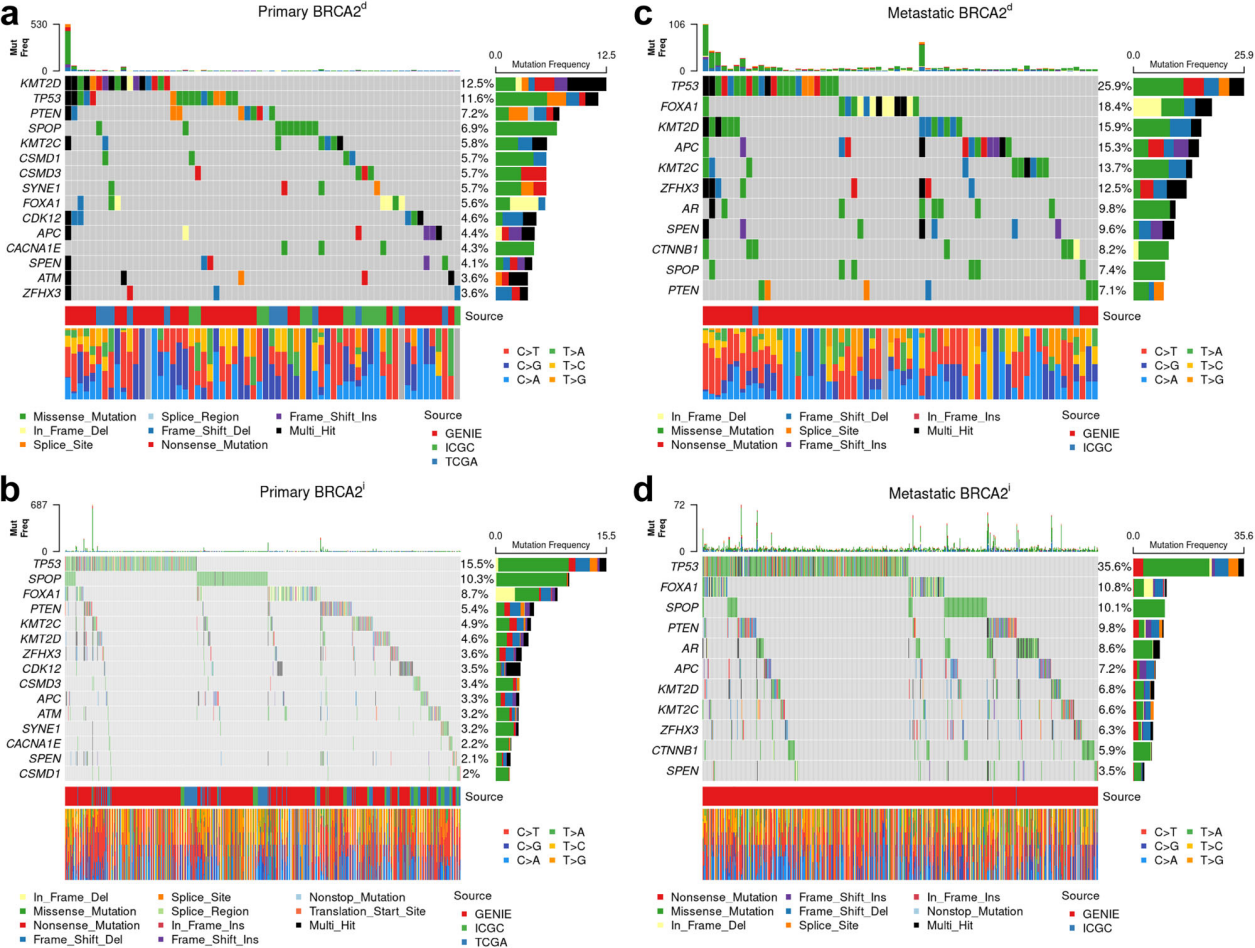
Oncoplots of pathogenic single nucleotide variants among primary and metastatic *BRCA2*-deficient and *BRCA2*-intact tumors. Plots correspond to (**a**) primary *BRCA2*-deficient tumors, (**b**) primary *BRCA2*-intact tumors, **c** metastatic *BRCA2*-deficient tumors, and **d** metastatic *BRCA2*-intact tumors. Figures display the most frequently altered candidate genes only.

The most common pathogenic SNVs among *BRCA2*^*d*^ and *BRCA2*^*i*^ primary tumors from the combined ICGC and GENIE data are shown in Fig. 3a, b. *KMT2D* had the highest pathogenic SNV frequency among primary *BRCA2*^*d*^ tumors (12.5%) with a significantly higher frequency among *BRCA2*^*d*^ tumors than *BRCA2*^*i*^ tumors (4.6%, FDR-adjusted *p* [*p*_adj_] = 0.0004). *TP53* had the second highest pathogenic SNV frequency among *BRCA2*^*d*^ tumors, but its frequency did not differ between *BRCA2*^*d*^ (11.6%) and *BRCA2*^*i*^ (15.5%) primary tumors (*p*_adj_ = 0.27) (Fig. 3a, b, Supplementary Table 4). Pathogenic SNVs at *PTEN* (7.2%), *SPOP* (6.9%), *KMT2C* (5.8%), *CSMD1* (5.7%), *SYNE1* (5.7%), *CSMD3* (5.7%), and *FOXA1* (5.6%) were also present in ≥5% of primary *BRCA2*^*d*^ tumors; however, these genes also did not differ in mutation frequency by tumor *BRCA2* status after multiple testing correction. When evaluating total SNV frequency, SNVs at 11 genes were significantly more frequent among primary *BRCA2*^*d*^ tumors, while none occurred at higher frequency among *BRCA2*^*i*^ tumors (Supplementary Table 5) (*p*_adj_ < 0.05). Of these genes, *CSMD3* was the most commonly altered among *BRCA2*^*d*^ tumors (67.1%), followed by *LRP1B* (66.2%), and *CSMD1* (64.3%). The total SNV frequency for *KMT2D* was also significantly higher for *BRCA2*^*d*^ (13.2%) than for *BRCA2*^*i*^ tumors (5.8%, *p*_adj_ = 0.003). Pathogenic SNVs in oncogenic pathways were not differentially enriched between *BRCA2*^*d*^ and *BRCA2*^*i*^ tumors. (Supplementary Table 6). Finally, the mean total and pathogenic transitions and transversions per patient did not differ by *BRCA2* status (Supplementary Fig. 1).

Among metastatic tumors from GENIE, *TP53* had the highest pathogenic SNV frequency in both *BRCA2*^*d*^ and *BRCA2*^*i*^ tumors (25.9% vs. 35.6%, *p*_adj_ = 0.07) (Fig. 3c, d, Supplementary Table 7). *FOXA1* (18.4%), *KMT2D* (15.9%), *APC* (15.3%), *KMT2C* (13.7%), and *ZFHX3* (12.5%) had pathogenic SNVs in ≥10% of metastatic *BRCA2*^*d*^ tumors. No gene differed in pathogenic SNV frequency by *BRCA2* status following FDR correction. The pathogenic SNV frequency at *SPOP* was 7.4% for *BRCA2*^*d*^ tumors and 10.1% for *BRCA2*^*i*^ tumors (*p*_adj_ = 0.45). When evaluating total SNV frequency, SNVs were enriched at *KMT2D* (17.1% vs. 6.9%), *RAD51B* (8.0% vs. 0.7%), *BRIP1* (6.1% vs. 1.0%), *BRCA1* (5.9% vs. 0.9%), and *RAD50* (3.8% vs. 0.1%) in metastatic *BRCA2*^*d*^ tumors relative to metastatic *BRCA2*^*i*^ tumors (all *p*_adj_ < 0.05) (Supplementary Table 8). Other genes with an SNV frequency ≥ 10% among metastatic *BRCA2*^*d*^ tumors include *TP53* (28.2%), *FOXA1* (18.4%), *APC* (16.5%), *KMT2C* (16.4%), *ZFHX3* (14.1%) and *GRIN2A* (11.1%), but SNV frequency did not significantly differ by *BRCA2* status for these genes.

Pathogenic SNV frequency did not significantly differ after multiple testing correction for any single gene between primary and metastatic *BRCA2*^*d*^ tumors in GENIE (Supplementary Table 9). Pathogenic SNVs at *FOXA1* were nominally less frequent among primary *BRCA2*^*d*^ tumors (5.5%) than metastatic *BRCA2*^*d*^ tumors (19.4%; *p*_adj_ = 0.03), as were pathogenic SNVs at *AR* (1.5% vs.10.3%; *p*_adj_ = 0.04).

We evaluated Catalog of Somatic Mutations in Cancer (COSMIC) signature similarity in *BRCA2*^*d*^ and *BRCA2*^*i*^ primary tumors (Supplementary Table 10)^17^. The only unique signature identified in *BRCA2*^*d*^ tumors was defective homologous recombination DNA damage repair (ID6). In both *BRCA2*^*d*^ and *BRCA2*^*i*^, COSMIC signatures were identified related to spontaneous deamination of 5-methylcytosine (clock-like signature; SBS1) and slippage during DNA replication of the replicated DNA strand (ID1 in both *BRCA2*^*d*^ and *BRCA2*^*i*^; ID2 in *BRCA2*^*i*^ only). We infer these to be signatures associated with prostate cancer, and not specific to *BRCA2*^*d*^ prostate tumors. Finally, we identified a signature similar to defective DNA mismatch repair among *BRCA2*^*i*^ tumors only.

### Copy number alteration (CNA) analyses

**Fig. 4:**
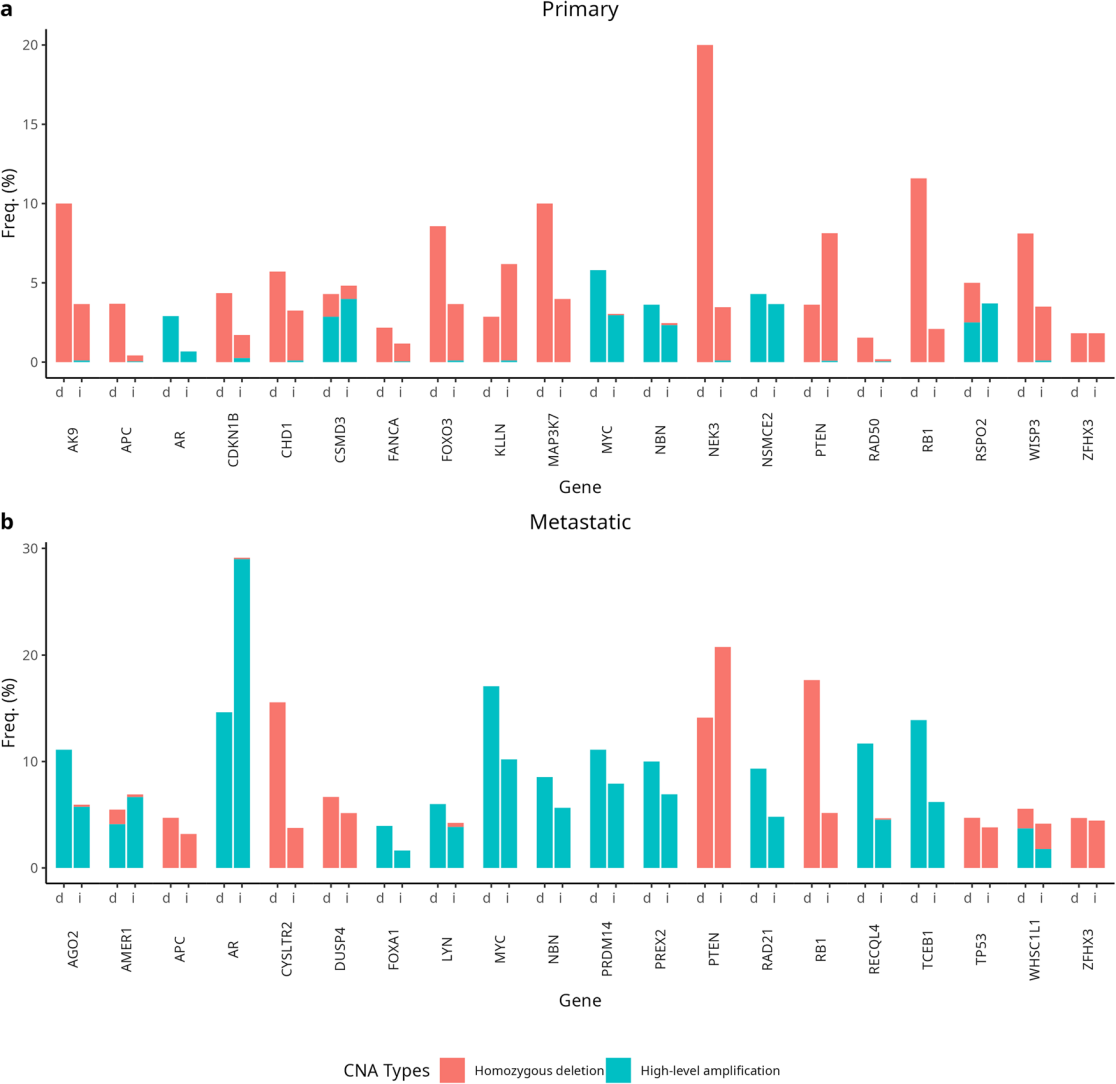
Pathogenic copy number variant frequency among *BRCA2*-deficient and *BRCA2*-intact tumors. Plots correspond to (**a**) primary and (**b**) metastatic tumors. Figures displays the most commonly altered candidate genes only. *d* = *BRCA2*-deficient; *i* = *BRCA2*-intact.

In primary tumors from the GENIE and TCGA datasets, homozygous deletions were significantly more frequent at three genes in *BRCA2*^*d*^ tumors relative to *BRCA2*^*i*^ tumors after multiple testing correction: *NEK3* (20.0% vs 3.4%), *RB1* (11.6% vs 2.1%), and *APC* (3.8% vs 0.4%) (Fig. 4a, Supplementary Table 11). Amplifications at *MYC* (5.8%), *NSMCE2* (4.3%), *NBN* (3.6%), and *AR* (2.9%) were the most common high-level amplifications among *BRCA2*^*d*^ primary tumors, but no gene differed in high-level amplification frequency by *BRCA2* status. Frequencies of low-level gains and LOH in primary tumors by *BRCA2* status are presented in Supplementary Table 10.

In metastatic tumors, the frequency of homozygous deletions at *RB1* was significantly higher among *BRCA2*^*d*^ than *BRCA2*^*i*^ tumors (17.7% versus 5.2%, *p*_adj_ = 0.0001) (Fig. 4b, Supplementary Table 12). No other single gene difference in homozygous deletion frequency by *BRCA2* status after multiple testing correction. The frequency of *PTEN* homozygous deletions was non-significantly lower among *BRCA2*^*d*^ tumors (14.1%) than *BRCA2*^*i*^ tumors (20.8%). Homozygous deletions at *CHD1* also did not differ by *BRCA2* status (5.7% *BRCA2*^*d*^ vs 3.1% *BRCA2*^*i*^, *p*_adj_ = 0.28). As was observed in primary tumors, no gene differed in high-level amplification frequency by *BRCA2* status in metastatic tumors. Among genes with high-level amplification frequencies ≥ 10% among *BRCA2*^*d*^ tumors, amplifications at *MYC*, *TCEB1*, *RECQL4*, *AGO2*, *PRDM14*, and *PREX2* were non-significantly more common among *BRCA2*^*d*^ tumors, while *AR* was non-significantly more commonly amplified among *BRCA2*^*i*^ tumors. Frequencies of low-level gains and LOH in metastatic tumors by *BRCA2* status are presented in Supplementary Table 12.

The frequency of homozygous deletions at *RB1* was nominally higher among metastatic *BRCA2*^*d*^ tumors (16.1%) than primary *BRCA2*^*d*^ tumors (4.4%, *p*_adj_ = 0.03) in the GENIE data, as was the frequency of homozygous deletions at *PTEN* (12.4% vs. 1.5%, *p*_adj_ = 0.01) (Supplementary Table 13). However, these frequencies were not significantly different following FDR correction. Likewise, no single gene differed in high-level amplification frequency between primary and metastatic *BRCA2*^*d*^ tumors, although amplifications at *AR* were nominally more frequent in metastatic tumors (15.4% vs. 4.4%, *p*_adj_ = 0.03).

### Structural variant (SV) analysis

The frequencies of *TMPRSS2-* and ETS-related SVs were estimated in the TCGA and GENIE data. In addition to 848 SVs that affected *TMPRSS2* and/or ETS family genes, 33 SVs affected a gene within 2Mbp of *TMPRSS2* or an ETS family gene and were assumed to impair function of that gene (Supplementary Table 14). The frequency of re-annotated SVs that were inferred to affect *TMPRSS2* or an ETS family gene did not differ by tumor *BRCA2* status. The frequency of *TMPRSS2-*ETS fusions was similar between primary *BRCA2*^*d*^ (32.8%) and *BRCA2*^*i*^ (31.1%) tumors (*p* = 0.78) (Table 1), as was the frequency of *TMPRSS2*-*ERG* fusions (28.4% vs 29.9%, p-0.62). Similarly, no differences in the frequency of *TMPRSS2-ETV1, TMPRSS2-*intragenic, or other SVs involving ETS genes were observed between *BRCA2*^*d*^ and *BRCA2*^*i*^ primary tumors.

**Table 1:** Structural variant (SV) frequency by tumor *BRCA2* alteration status for primary and metastatic prostate tumors in GENIE and TCGA.

		Primary tumors					Metastatic tumors				
SV Type	SV Presence	*n* *BRCA2*^*d*^	%	*n* *BRCA2*^*i*^	%	*P*-value	n *BRCA2*^*d*^	%	n *BRCA2*^*i*^	%	*P*-value^a^
*TMPRSS2*-ETS	Present	22	32.8%	533	31.1%	0.78	17	25.0%	220	27.2%	0.78
	Absent	45	67.2%	1181	68.9%		51	75.0%	588	72.8%	
*ETS-*Other	Present	0	0.0%	28	1.5%	0.62	0	0.0%	3	0.4%	1.00
	Absent	70	100.0%	1853	99.8%		68	100.0%	805	99.6%	
*TMPRSS2-ERG*	Present	19	28.4%	513	29.9%	0.89	15	22.1%	212	26.4%	0.48
	Absent	48	71.6%	1201	70.1%		53	77.9%	592	73.6%	
*TMPRSS2*-*ETV1*	Present	1	1.5%	4	0.2%	0.17	2	3.0%	1	0.1%	0.017
	Absent	66	98.5%	1708	99.8%		65	97.0%	803	99.9%	
*TMPRSS2*-intragenic	Present	2	3.0%	32	1.9%	0.37	1	1.5%	23	2.9%	1.00
	Absent	65	97.0%	1682	98.1%		67	98.5%	781	97.1%	

^a^*P*-values based on a two-sided Fisher’s exact test.

In metastatic tumors, *TMPRSS2*-*ETV1* fusions were more common among *BRCA2*^*d*^ (3.0%) than *BRCA2*^*i*^ tumors (0.1%; *p* = 0.017). In metastatic tumors, the prevalence of *TMPRSS2*-ETS fusions did not differ by tumor *BRCA2* status, nor did the prevalence of *TMPRSS2-ERG, TMPRSS2-*intragenic or other SVs involving ETS family genes.

## Discussion

There is mounting evidence that the molecular signatures of prostate tumors in men with *BRCA2*^*d*^ prostate tumors either from germline or somatic mutation exhibit a different molecular signature relative to *BRCA2*^*i*^. Our results confirm some prior reports of mutational patterns in *BRCA2*^*d* ^^18,19^, but also identify new mutational patterns in *BRCA2*^*d*^ prostate tumors in part because of the increased sample size of our dataset.

We report that the TMB of SNVs in *BRCA2*^*d*^ tumors is significantly higher than in *BRCA2*^*i*^ tumors, in line with prior findings that the somatic mutation rate is 2.9-fold higher in high-grade *BRCA2*^*d*^ prostate tumors^20^. However, we found no significant difference in pathogenic SNV TMB between *BRCA2*^*d*^ and *BRCA2*^*i*^ tumors. We also observed that pathogenic and total SNV frequencies at *KMT2D* were higher in *BRCA2*^*d*^ primary tumors than *BRCA2*^*i*^ tumors. *KMT2D* encodes a histone methyltransferase that methylates the Lys-4 position of histone H3, and is a member of the ASCOM protein complex, which has been shown to be a transcriptional regulator of the beta-globin and estrogen receptor genes. *KMT2D* is associated with activation of *PKN1*, which stimulates transcription of the AR-regulated kallikrein genes *KLK2* and *KLK3*^21^*. KMT2D* has been reported to be highly mutated in prostate tumors, and high *KMT2D* transcription is associated with poor prostate cancer prognosis. *KMT2D* epigenetically activates PI3K/AKT pathway and epithelial-mesenchymal transition by targeting *LIFR* and *KLF4* and thus serves as a putative therapeutic target for prostate cancer^22^. Our observation that *KMT2D* is more commonly mutated in *BRCA2*^*d*^ suggests that it plays a role in *BRCA2*-associated prostate carcinogenesis and may identify therapeutic targets for *BRCA2*^*d*^ prostate cancer.

*BRCA2* plays a critical role in the regulation of homologous recombination (HR) repair of double-stranded DNA breaks^23^, and protein partners involved in this process have been described. We identified elevated SNV frequencies in known *BRCA2*-interacting pathway genes *RAD51B*, *BRIP1*, *BRCA1*, and *RAD50* in metastatic *BRCA2*^*d*^ tumors relative to *BRCA2*^*i*^ tumors. These results confirm the role of these loci in *BRCA2*-associated pathways involved in prostate carcinogenesis and suggest that they are also involved in *BRCA2*^*d*^ prostate tumorigenesis and progression. This observation is consistent with reports of mutation signatures associated with HR defects in *BRCA2* prostate tumors that do not have *BRCA2* germline PSV^24^. We did not observe differences in *SPOP* mutation or *CHD1* loss frequency by tumor *BRCA2* status, although these genes have been implicated in impaired HR repair^25–27^.

We identified higher homozygous deletion frequencies for *NEK3*, *RB1*, and *APC* in *BRCA2*^*d*^ primary tumors, and for *RB1* deletions in metastatic *BRCA2*^*d*^ tumors. Chromosome 13q14, which contains *RB1* (chromosome 13q14.2) and *NEK3* (chromosome 13q14.3), is among the most commonly altered loci in prostate tumors, with *RB1* and *BRCA2* being commonly co-deleted in prostate tumors^28,29^. In human prostate tumor cell line models, concomitant *BRCA2* and *RB1* loss induces an epithelial-to-mesenchymal transition, resulting in a more aggressive tumor phenotype^30^. Alterations at this locus are associated with poor prognosis and unfavorable tumor characteristics^31,32^. Our observations suggest that not only is this locus important for prostate carcinogenesis and for prediction of poor prognosis, it may also in part explain the difference in prostate tumor aggressiveness and poor prognosis seen in *BRCA2*^*d*^ prostate cancer.

CNA in prostate tumors among carriers of *BRCA2* PSV have been reported to be 3-fold higher than in tumors without *BRCA2* PSV, with copy number gains being more common in the region encompassing *c-MYC*^13^. While *c-MYC* amplifications were among the most common events observed in our dataset, we did not identify a significant excess in *BRCA2*^*d*^ compared with *BRCA2*^*i*^. This observation may indicate different effects of *BRCA2* PSV compared with *BRCA2*^*d*^ tumors, which were not exclusively associated with germline PSV.

*APC* is a tumor suppressor gene that activates elements of the Wnt/β-catenin signaling pathway, and is mutated in 3-10% of prostate tumors^33^. *APC* is involved in phosphorylation of β-catenin and subsequent degradation. Knowledge of this pathway has led to the development of Wnt signaling inhibitors. *APC* promoter methylation exists at high levels in prostate tumors and is a poor prognosis indicator in prostate cancer^34^. While we did not have data to address *APC* promoter methylation, we report 3.8% *APC* deletions in primary *BRCA2*^*d*^ compared with 0.4% in *BRCA2*^*i*^. These data are consistent with elevated levels of *APC* inactivation in *BRCA2*^*d*^. The lower proportion of *APC* mutations reported here in *BRCA2*^*i*^ tumors compared with other reports in the literature is due to our association of *APC* CNA only, and not other mutational types or promoter methylation. This result builds upon the findings of Taylor et al. who observed that amplifications of the Wnt/β-catenin pathway modulators *MED12* and *MED12L* were also more common among *BRCA2* PSV carriers and that *BRCA2*^*d*^ prostate cancers have been shown to experience global hypomethylation relative to sporadic cancers^7^.

Fusion proteins involving *TMPRSS2*, particularly those involving *ERG* and other ETS family members, are common SV alterations in prostate tumors. *TMPRSS2*-ETS fusions, and *TMPRSS2-ERG* fusions specifically, were common in both *BRCA2*^*d*^ and *BRCA2*^*i*^, but we did not identify a difference in the frequency of these events by *BRCA2* status in either primary or metastatic tumors, as has been reported for germline *BRCA2*^*d*^ prostate cancer^7,35,36^. We observed a subset of SVs which affected genes in close proximity to *TMPRSS2* or ETS family genes and were assumed to impact the function of the neighboring gene. Given the low frequency of this occurrence, inclusion or exclusion of these SVs inferred to be *TMPRSS2*-ETS fusions did not substantially affect estimates of SV frequencies. However, misclassification of functional *TMPRSS2* and/or ETS family genes may exist if consideration of nearby mutated genes are not considered. This observation is dependent on the interval considered. We were also not able to assess the effects of these SV events on gene function with the data available. We did observe a significantly elevated frequency of *TMPRSS2-ETV1* fusions in metastatic *BRCA2*^*d*^ tumors compared to *BRCA2*^*i*^. *ETV1* is a target of the androgen receptor (AR). *ETV1* and *AR*, interact in prostate tissue to regulate cell invasion^37^. Decreased ETV1 expression disrupts the ability of both androgen-dependent and androgen-independent prostate cell invasion, independent of *TMPRSS2* fusion.

Our analysis represents the largest to date of primary and metastatic *BRCA2*^*d*^ prostate tumors and examined signatures across multiple classes of somatic mutation. While we report biologically plausible associations between somatic mutations and *BRCA2*^*d*^ and *BRCA2*^*i*^ prostate tumors, our analysis is limited in a number of ways. First, while it is well known that germline *BRCA2* PSV are associated with more aggressive disease, we were not able to evaluate how somatic mutational events were correlated with clinical traits. We used a surrogate comparison of primary vs. metastatic tumors to compare mutations in these two tumor groups, but this is not an adequate surrogate for the severity analyses that may be required to guide inferences about prognosis or management of disease. We applied multiple tools to ascertain *BRCA2*-deficiency^38–40^; however, the potential for misclassification of *BRCA2*^*d*^ or *BRCA2*^*i*^ tumors remains and use of alternative tools may have led to different designations of tumor *BRCA2*-deficiency^41^. Such misclassification would likely attenuate differences in alteration patterns between *BRCA2*^*d*^ or *BRCA2*^*i*^ tumors. Our analysis maximized the sample size available by integrating data across multiple publicly available sources, including TCGA, ICGC, and GENIE. These sources use different platforms and approaches for variant calling. As a result, misclassification of alterations likely differs across the sources, but we do not expect that the extent of misclassification differed for *BRCA2*^*d*^ and *BRCA2*^*i*^ tumors. In our efforts to account for potential misclassification, we noted that the annotations and platforms used (e.g., panel vs. whole exome/genome sequencing) could in some cases not be harmonized. Therefore, we harmonized only those data that could be combined in any single analysis (Fig. 1).

Our results provide evidence that somatic mutational patterns in prostate tumors may in part explain why *BRCA2*^*d*^ tumors have more aggressive characteristics than *BRCA2*^*i*^ tumors and focus attention on novel molecular events and pathways that may be used to understand the unique etiology of *BRCA2*^*d*^ tumors. We identify Wnt/β-catenin, PI3K, and homologous double-stranded break repair pathways as hallmarks of *BRCA2*^*d*^ prostate tumors. These patterns suggest great potential for molecularly targeted screening, monitoring, therapeutic development, and clinical management of these tumors.

## Methods

### Study sample and data processing

De-identified mutation files for SNVs, CNAs, and SVs and clinical data from the International Cancer Genome Consortium (ICGC) PRAD-CA, PRAD-CN, PRAD-FR, and PRAD-UK projects were downloaded from the ICGC Data Portal (https://dcc.icgc.org/releases/release_28)^14^. The American Association for Cancer Research (AACR) Project Genomics Evidence Neoplasia Information Exchange (GENIE) de-identified SNV, CNA, and SV alteration and clinical data for prostate adenocarcinomas were retrieved from Sage Bionetworks Synapse platform—release 8 (https://www.synapse.org)^15^. De-identified SNV, CNA, SV, and clinical data for prostate adenocarcinomas in the TCGA PanCancer Atlas were retrieved from cBioportal^16^. The ICGC and GENIE data were filtered to retain only primary and metastatic samples. The ICGC SNVs were processed using *maftools* to obtain a common data format as SNVs from GENIE and TCGA^42^. GENIE CNAs were obtained in CNA formats that indicated a gene was disrupted in a specific tumor sample. GENIE data were further filtered to retain only samples with available SNV, CNA, and SV profiling unless the sample had an alteration affecting BRCA2. The eight retained panels for *BRCA2*^*i*^ samples were: COLU-CCCP-V1, DFCI-ONCOPANEL-1, MSK-IMPACT341, MSK-IMPACT410, MSK-IMPACT468, VICC-01-T5A, VICC-01-T7, VICC-01-DX1). The panels included for *BRCA2*^*d*^ samples include DFCI-ONCOPANEL-2, DFCI-ONCOPANEL-3, DFCI-ONCOPANEL-3.1, DUKE-F1-T7, GRCC-MOSC4, GRCC-MOSC3, UHN-555-PROSTATE-V1, UHN-555-V1, and YALE-OCP-V3, in addition to the eight used for *BRCA2*^*i*^ samples. This study is compliant with all relevant ethical regulations and was deemed exempt by the institutional review board of Dana-Farber Cancer Institute (Protocol #19-236). The GENIE, ICGC, and TCGA consortia were responsible for obtaining patient informed consent or waivers of consent.

All samples from ICGC, TCGA, and GENIE were systematically reviewed by pathologists to confirm the histopathologic diagnosis and to ensure tumor cellularity met required thresholds defined by dataset and contributing institution. Data regarding patient receipt of neoadjuvant therapy prior to tumor resection were not available for primary tumors in GENIE and ICGC, while 99.6% of patients in TCGA, which contains primary tumors only, did not receive neoadjuvant therapy.

The analytic sample set is summarized in a flow diagram in Fig. 1. A total of 4298 patients with prostate adenocarcinomas were identified across ICGC (*n* = 703), GENIE (*n* = 3101), and TCGA (*n* = 494). Samples with only blood and non-tumor tumor tissue data were excluded from the analyses, as were samples for two female patients. GENIE samples for which *BRCA2* alterations were not profiled were excluded from analysis. If multiple primary or metastatic samples were available for a given patient, a single sample was selected using the following hierarchy: (1) the sample where the *BRCA2* PSV was detected was selected, (2) the sample with maximal available genomic data types was selected (e.g., a sample with SNV and CNA profiling was prioritized over a sample with SNV profiling only), (3) a sample was randomly selected if neither of the first two criteria was fulfilled. Analyses of SNVs were conducted in a harmonized merged ICGC, GENIE, and TCGA dataset. ICGC CNA and SV could not be adequately harmonized with the corresponding GENIE and TCGA data. Thus, CNA and SV analyses were limited to the GENIE and TCGA data. Sample sizes of the final analytic population by dataset and genomic data type for primary and metastatic tumors are shown in Fig. 1.

### Identification of BRCA2-deficient tumors

*BRCA2*^*d*^ status was determined by assessing the presence of SNV, CNA, and SV involving *BRCA2* in tumors. Deep deletions or amplifications of *BRCA2* were assumed to result in impaired *BRCA2* function, while tumors with LOH or low-level gains were considered *BRCA2*^*i*^. Likewise, patients with SV affecting *BRCA2* were classified as *BRCA2*^*d*^. The potential impact of somatic SNV on *BRCA2* function was evaluated by variant type and consequence using OncoKB, SnpEff, and ClinVar^38–40^. GENIE data were restricted to panels that profiled SNVs, CNAs, and SVs. Accordingly, *BRCA2*^*i*^ samples in GENIE and TCGA were known to not harbor any SNV, CNA, or SV that affected *BRCA2*. In order to preserve an adequate sample size in ICGC, no analogous restrictions regarding profiling were required. *BRCA2*^*i*^ samples in ICGC did not have any SNV, CNA, or SV profiling of *BRCA2*. A summary of patients with *BRCA2* PSV and PSVs is provided in Supplementary Table 1.

### Single nucleotide variant (SNV) analyses

Sixty-seven candidate genes were identified for inclusion in somatic mutation analyses from the literature^10,11,43,44^. These included 18 hereditary prostate cancer genes, 21 genes that are known interactors with *BRCA2*, and 28 genes that are recurrently mutated in prostate cancer (Supplementary Table 2). The a priori identified candidate genes were supplemented with genes that had among the top 20 highest pathogenic SNV frequencies in the ICGC, GENIE, and TCGA data for primary or metastatic samples. SNV frequencies were defined as the proportion of individuals with profiling of a particular gene who harbored a variant affecting the gene of interest. The sample size for frequency calculations varied across genes, as not all candidate genes were profiled in the GENIE panels. To ensure sufficient power, only genes profiled in at least 40% of tumors were carried forward for analysis. 76 total candidate genes were studied in primary tumors and 51 total candidate genes in metastatic tumors. SNV frequencies were calculated including all mutations and for likely pathogenic mutations only based on Sequence Ontology and the resulting predicted impact by Ensembl (http://uswest.ensembl.org/info/genome/variation/prediction/predicted_data.html). SNV frequencies were visualized using modified Oncoplots, accounting for the variable gene profiling coverage across individuals^42^. Differences in candidate gene mutation frequency by *BRCA2* alteration status and between primary and metastatic *BRCA2*^*d*^ tumors in the GENIE data were evaluated.

Enrichment of oncogenic pathways was evaluated using *maftools* in primary tumors from TCGA and ICGC^42,45^. In addition to the eight pathways pre-selected in *maftools*, a chromatin remodeling pathway was included and consisted of: *SWI1*, *SWI2*, *SNF2*, *SWI3*, *SWI5*, *SWI6*, *HDAC1*, *HDAC2*, *RbAp46*, *RbAp48*, *MTA1*, *MTA2*/*MTA3*, *MBD3*, *MBD2*, *CHD3*, *CHD4*, *INO80*, and *SWR1*. The proportion of samples harboring a pathogenic SNV within the pathway of interest was compared by tumor *BRCA2* status.

Tumor mutational burden (TMB) was calculated using SNV data for primary tumors for the ICGC data with whole-genome sequencing (WGS) profiling. Data from TCGA and GENIE, which used whole-exome sequencing (WES) and targeted panels, respectively, were not included to ensure comparability of TMB measures across sites. TMB was defined as the total number of somatic mutations present in a tumor sample per megabase (Mb)^46^. Total TMB captured all SNVs regardless of gene or variant type. Pathogenic TMB was limited to variants in known genes meeting pathogenicity criteria (http://uswest.ensembl.org/info/genome/variation/prediction/predicted_data.html). The capture size for the WGS datasets was set to 3000 Mbp. Differences in pathogenic TMB and total TMB by tumor *BRCA2* status were assessed.

The number of total and pathogenic transitions and transversions were calculated for patients with primary tumors in ICGC with WGS SNV profiling. Differences in the number of total and pathogenic transitions and transversions by tumor *BRCA2* status were assessed.

Enrichment for Catalog of Somatic Mutations in Cancer (COSMIC) single base substitution (SBS), doublet base substitution (DBS), and small insertion and deletion (ID) mutational signatures was performed for primary *BRCA2*^*d*^ and *BRCA2*^*i*^ tumors using SigMiner in the ICGC and TCGA data using the default parameters^17,47^. SNV data were converted to maf format using *maftools* and a matrix was constructed for non-negative matrix factorization decomposition using the sig_tally function. The optimal number of signatures was automatically extracted using the sig_auto_extract function. Signatures with cosine similarity >0.9 and known etiologies were reported for *BRCA2*^*d*^ and *BRCA2*^*i*^ tumors.

### Copy number alteration analysis

CNA analyses were conducted using data from GENIE (primary and metastatic) and TCGA (primary). Candidate genes for CNA analyses were identified in a similar manner as for the SNV analyses. 67 genes identified in the literature were supplemented with genes among the top 20 most commonly copy number-altered in primary or metastatic tumors (Supplementary Table 2). Only genes profiled in at least 40% of individuals were carried forward for analysis, resulting in 75 candidate genes for primary tumors and 57 candidate genes for metastatic tumors. CNA frequencies were defined as the proportion of individuals with profiling of a particular gene who harbored a CNA affecting the gene of interest. Differences in candidate gene CNA frequency by *BRCA2* alteration status were evaluated using Fisher’s exact test separately for primary and metastatic samples. The frequency of pathogenic CNAs was also compared between primary and metastatic *BRCA2*^*d*^ tumors in the GENIE data.

### TMPRSS2 and ETS structural variant (SV) analyses

Structural variant (SV) analyses were conducted in GENIE and TCGA samples (Fig. 1) to investigate the association between *BRCA2* deficiency and the occurrence of *TMPRSS2*- and ETS-related SVs. Alteration frequencies were calculated for harboring any SV as well as for SVs affecting *TMPRSS2*, *ERG*, or ETS family genes, accounting for gene coverage across the GENIE panels. Samples with SV profiling for at least one ETS family gene were included in estimating frequencies of ETS family SVs. ETS genes considered included: *ELF1*, *ELF2*, *ELF3*, *ELF4*, *ELF5*, *ELK1*, *ELK3*, *ELK4*, *ERF*, *ERG*, *ESE3*, *ETS1*, *ETS2*, *ETV1*, *ETV2*, *ETV3*, *ETV4*, *ETV5*, *ETV6*, *ETV7*, *GABPA*, *FLI1*, *FEV*, *SPDEF*, *SPI1*, *SPIB*, and *SPIC*. To account for potential misannotation of SVs, SVs involving neighboring genes within 2mbp of *TMPRSS2* or an ETS family gene were classified as affecting *TMPRSS2* or the ETS gene, as appropriate. SVs of interest were classified as *TMPRSS2*-ETS or ETS-other. Differences in alteration frequencies for these two SV categories by *BRCA2* alteration status as well as differences in the frequency of *TMPRSS2-ERG*, *TMPRSS2-ETV1*, and *TMPRSS2*-intragenic SVs were tested.

### Hypothesis testing

Distributions of clinical covariates, SNVs, CNAs, and SVs by *BRCA2* alteration status were compared using *t*-tests or Wilcoxon rank-sums tests for continuous variables and Fisher’s exact test for categorical variables. The Benjamini–Hochberg procedure was used to control the false discovery rate (FDR ≤ 5%)^48^.

### Reporting summary

Further information on research design is available in the Nature Research Reporting Summary linked to this article.

## Supplementary information

Supplementary Tables and Figures

REPORTING SUMMARY

## Data Availability

The data that support the findings of this study are available from the AACR Project GENIE (https://www.synapse.org/#!Synapse:syn7222066/wiki/405659), the International Cancer Genome Consortium (https://dcc.icgc.org/releases/release_28), and The Cancer Genome Atlas (https://www.cbioportal.org/study/summary?id=prad_tcga_pan_can_atlas_2018).

## Code availability

Data analysis was conducted using R (version 4.0.5) and Python (version 3.8.5). Maftools (version 2.8.05), sigminer (version 2.1.1), and custom scripts were used to complete the analyses. Code is available upon request.
