# Supplementary material for: The tumor mutational landscape of BRCA2-deficient primary and metastatic prostate cancer
Source: NPJ Precis Oncol. 2022 Jun 17;6:39. doi: 10.1038/s41698-022-00284-6 (PMC9205939; doi:10.1038/s41698-022-00284-6)
Supplement: Supplementary file 1 — Supplementary Tables and Figures [file 41698_2022_284_MOESM1_ESM.docx]

**The Tumor Mutational Landscape of *BRCA2*-deficient Primary and Metastatic Prostate Cancer**

**Supplementary Tables and Figures**

[**Supplementary Figure 1** 2](#_Toc100132643)

[**Supplementary Table 1** 3](#_Toc100132644)

[**Supplementary Table 2** 12](#_Toc100132645)

[**Supplementary Table 3** 14](#_Toc100132646)

[**Supplementary Table 4** 15](#_Toc100132647)

[**Supplementary Table 5** 17](#_Toc100132648)

[**Supplementary Table 6** 19](#_Toc100132649)

[**Supplementary Table 7** 20](#_Toc100132650)

[**Supplementary Table 8** 22](#_Toc100132651)

[**Supplementary Table 9** 24](#_Toc100132652)

[**Supplementary Table 10** 26](#_Toc100132653)

[**Supplementary Table 11** 27](#_Toc100132654)

[**Supplementary Table 12** 35](#_Toc100132655)

[**Supplementary Table 13** 43](#_Toc100132656)

[**Supplementary Table 14** 45](#_Toc100132657)

**Supplementary Figure 1**. Distribution of the number of transitions and tumors in primary tumors from ICGC by tumor BRCA2 status. Panels display distributions of: **(A)** total transitions, **(B)** total transversions, **(C)** pathogenic transitions, and **(D)** pathogenic transversions. Differences in the distribution by BRCA2 status were assessed using the Wilcoxon rank-sum test. Box plots show from top to bottom: maximum, third quartile, median, first quartile, minimum.


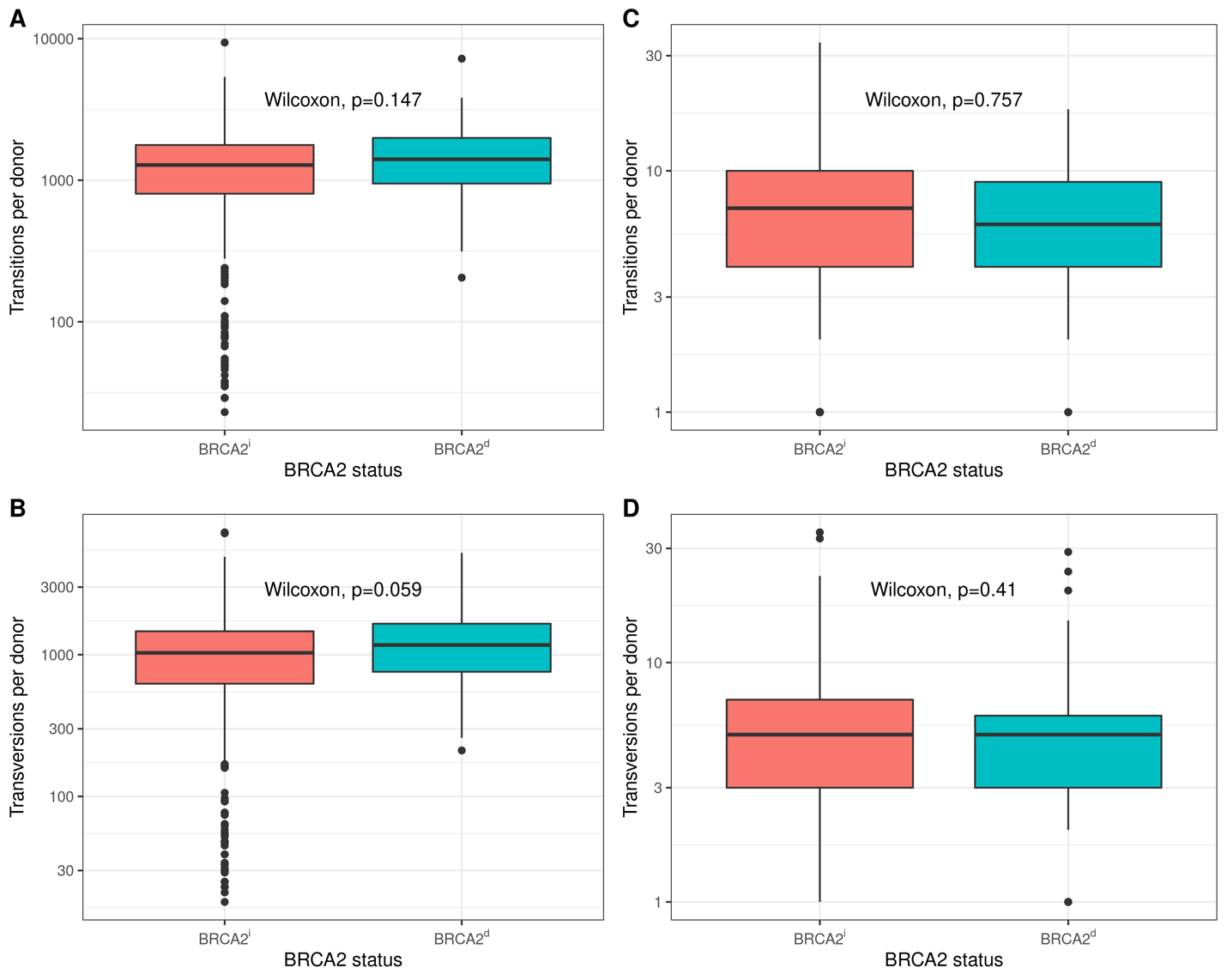


**Supplementary Table 1**. List of BRCA2-deficient donors

| **Patient ID** | **Data Source** | **Tumor Sample Barcode** | **Sample Type** | **SNV Profiling** | **CNA Profiling** | **SV Profiling** | **BRCA2 Alteration Type** | **BRCA2 Alteration Description** |
| --- | --- | --- | --- | --- | --- | --- | --- | --- |
| DO228619 | ICGC | SA595812 | Primary | yes | no | no | SV/CNA | BRCA2 intragenic variant, Deep Deletion |
| DO228657 | ICGC | SA595729 | Primary | yes | no | no | CNA | Deep Deletion |
| DO228686 | ICGC | SA595838 | Primary | yes | no | no | SV | BRCA2-N4BP2L2 fusion |
| DO228781 | ICGC | SA595976 | Primary | yes | no | no | CNA | Deep Deletion |
| DO229396 | ICGC | SA598816 | Primary | yes | no | no | CNA | Deep Deletion |
| DO229409 | ICGC | SA598810 | Primary | yes | no | no | CNA | Deep Deletion |
| DO229411 | ICGC | SA598922 | Primary | yes | no | no | CNA | Deep Deletion |
| DO229419 | ICGC | SA599001 | Primary | yes | no | no | CNA | Deep Deletion |
| DO229435 | ICGC | SA598972 | Primary | yes | no | no | SV | BRCA2 intragenic variant, non-pathogenic CNA |
| DO229449 | ICGC | SA598983 | Primary | yes | no | no | CNA | Deep Deletion |
| DO229490 | ICGC | SA598779 | Primary | yes | no | no | CNA | Amplification |
| DO229529 | ICGC | SA598970 | Primary | yes | no | no | CNA | Deep Deletion, non-pathogenic SNV (chr13:g.32902394->C) |
| DO229553 | ICGC | SA598771 | Primary | yes | no | no | CNA | Deep Deletion |
| DO229557 | ICGC | SA598847 | Primary | yes | no | no | CNA | Deep Deletion |
| DO229567 | ICGC | SA598910 | Primary | yes | no | no | CNA | Deep Deletion |
| DO229570 | ICGC | SA598905 | Primary | yes | no | no | CNA | Deep Deletion |
| DO229574 | ICGC | SA598785 | Primary | yes | no | no | SV | Fusion involving BRCA2 and unknown gene |
| DO229580 | ICGC | SA598829 | Primary | yes | no | no | CNA | Deep Deletion |
| DO229581 | ICGC | SA598893 | Primary | yes | no | no | CNA | Deep Deletion |
| DO229582 | ICGC | SA598926 | Primary | yes | no | no | CNA | Deep Deletion |
| DO229588 | ICGC | SA598918 | Primary | yes | no | no | CNA | Deep Deletion |
| DO230496 | ICGC | SA603697 | Primary | yes | no | no | CNA | Deep Deletion |
| DO230503 | ICGC | SA603299 | Primary | yes | no | no | CNA | Deep Deletion |
| DO230560 | ICGC | SA601282 | Primary | yes | no | no | CNA | Amplification |
| DO230589 | ICGC | SA603688 | Primary | yes | no | no | CNA | Deep Deletion |
| DO230592 | ICGC | SA603706 | Primary | yes | no | no | CNA | Deep Deletion |
| DO230597 | ICGC | SA603912 | Primary | yes | no | no | CNA | Deep Deletion |
| DO230633 | ICGC | SA603918 | Primary | yes | no | no | CNA | Deep Deletion |
| DO230637 | ICGC | SA603969 | Primary | yes | no | no | CNA | Deep Deletion |
| DO46788 | ICGC | SA506738 | Primary | yes | no | no | CNA | Deep Deletion |
| DO46790 | ICGC | SA506742 | Primary | yes | no | no | CNA | Deep Deletion |
| DO51044 | ICGC | SA530426 | Primary | yes | no | no | CNA | Deep Deletion |
| DO51047 | ICGC | SA530432 | Primary | yes | no | no | CNA | Deep Deletion |
| DO51049 | ICGC | SA530436 | Primary | yes | no | no | SNV/CNA | chr13:g.32968994->T, FS Insertion, D3142V?, Deep Deletion |
| DO51051 | ICGC | SA530440 | Primary | yes | no | no | CNA | Deep Deletion |
| DO51055 | ICGC | SA530448 | Primary | yes | no | no | CNA | Deep Deletion |
| DO51062 | ICGC | SA530462 | Primary | yes | no | no | CNA | Deep Deletion |
| DO51097 | ICGC | SA530468 | Primary | yes | no | no | CNA | Deep Deletion |
| DO51114 | ICGC | SA530532 | Primary | yes | no | no | CNA | Deep Deletion |
| DO51118 | ICGC | SA530546 | Primary | yes | no | no | CNA | Deep Deletion |
| DO51119 | ICGC | SA530550 | Primary | yes | no | no | CNA | Deep Deletion |
| DO51122 | ICGC | SA530562 | Primary | yes | no | no | CNA | Deep Deletion |
| DO51127 | ICGC | SA530583 | Primary | yes | no | no | CNA | Deep Deletion, non-pathogenic SNVs chr13:g.32895962->T unknown impact, chr13:g.32895963->T, unknown impact |
| DO51128 | ICGC | SA530587 | Primary | yes | no | no | CNA | Deep Deletion |
| DO51131 | ICGC | SA530598 | Primary | yes | no | no | CNA | Deep Deletion |
| DO51144 | ICGC | SA530626 | Primary | yes | no | no | CNA | Deep Deletion |
| DO51153 | ICGC | SA530644 | Primary | yes | no | no | CNA | Deep Deletion |
| DO51956 | ICGC | SA538912 | Metastasis | yes | no | no | CNA | Amplification |
| DO51958 | ICGC | SA538862 | Metastasis | yes | no | no | CNA | Amplification, non-pathogenic SNV chr13:g.32940066A>G unknown impact |
| DO51958 | ICGC | SA538917 | Primary | yes | no | no | CNA | Amplification, non-pathogenic SNV chr13:g.32940066A>G unknown impact |
| DO51962 | ICGC | SA538885 | Metastasis | yes | no | no | CNA/SV | Deep Deletion, BRCA2 intragenic variant, BRCA2-MRPS27 fusion, BRCA2-ATG10 fusion |
| DO51965 | ICGC | SA538918 | Metastasis | yes | no | no | CNA | Amplification |
| GENIE-DFCI-000893 | GENIE | GENIE-DFCI-000893-10091 | Primary | yes | yes | no | SNV | R1010Efs*33, non-pathogenic CNA |
| GENIE-DFCI-001119 | GENIE | GENIE-DFCI-001119-1914 | Primary | yes | yes | yes | SNV | F1866Lfs*8, non-pathogenic CNA |
| GENIE-DFCI-002984 | GENIE | GENIE-DFCI-002984-1962 | Primary | yes | yes | yes | SNV | D1911Vfs*50, non-pathogenic CNA |
| GENIE-DFCI-003054 | GENIE | GENIE-DFCI-003054-4162 | Primary | yes | yes | no | SNV | F1182*, non-pathogenic CNA |
| GENIE-DFCI-003715 | GENIE | GENIE-DFCI-003715-1451 | Primary | yes | yes | yes | SNV | E2924*, non-pathogenic CNA |
| GENIE-DFCI-004322 | GENIE | GENIE-DFCI-004322-4115 | Primary | yes | yes | no | SV | BRCA2-SMIM14 fusion, non-pathogenic CNA |
| GENIE-DFCI-007587 | GENIE | GENIE-DFCI-007587-4036 | Primary | yes | yes | no | SNV | L413Hfs*17, W2788*, non-pathogenic CNA |
| GENIE-DFCI-010493 | GENIE | GENIE-DFCI-010493-9767 | Primary | yes | yes | no | SNV | P222Lfs*8, non-pathogenic CNA |
| GENIE-DFCI-011472 | GENIE | GENIE-DFCI-011472-11033 | Metastasis | yes | yes | no | SNV | Q1291*, additional non-pathogenic SNV, non-pathogenic CNA |
| GENIE-DFCI-035420 | GENIE | GENIE-DFCI-035420-74293 | Primary | yes | yes | no | SNV | R2784Q, non-pathogenic CNA |
| GENIE-DFCI-036793 | GENIE | GENIE-DFCI-036793-74802 | Primary | yes | yes | no | CNA | Deep Deletion |
| GENIE-DFCI-038296 | GENIE | GENIE-DFCI-038296-218175 | Primary | yes | yes | no | SNV | Q2157Ifs*18, non-pathogenic CNA |
| GENIE-DFCI-038902 | GENIE | GENIE-DFCI-038902-188474 | Metastasis | yes | yes | no | SNV | N1626Sfs*12, non-pathogenic CNA |
| GENIE-DFCI-051157 | GENIE | GENIE-DFCI-051157-218559 | Primary | yes | yes | no | SNV | W2970*, non-pathogenic CNA |
| GENIE-DFCI-080253 | GENIE | GENIE-DFCI-080253-333000 | Metastasis | yes | yes | no | SV | BRCA2-TMPRSS2 fusion, non-pathogenic CNA |
| GENIE-DFCI-096989 | GENIE | GENIE-DFCI-096989-805293 | Metastasis | yes | yes | no | CNA | Deep Deletion |
| GENIE-DFCI-097462 | GENIE | GENIE-DFCI-097462-2210562 | Metastasis | yes | yes | no | SV/SNV | N1626Sfs*12, BRCA2 intragenic fusion |
| GENIE-DFCI-097462 | GENIE | GENIE-DFCI-097462-436964 | Primary | yes | yes | no | SNV | N1626Sfs*12, non-pathogenic CNA |
| GENIE-DFCI-097726 | GENIE | GENIE-DFCI-097726-805326 | Metastasis | yes | yes | no | CNA | Deep Deletion |
| GENIE-DFCI-098474 | GENIE | GENIE-DFCI-098474-853284 | Metastasis | yes | yes | no | SNV | Y989*, non-pathogenic CNA |
| GENIE-DFCI-105924 | GENIE | GENIE-DFCI-105924-436283 | Primary | yes | yes | no | SNV | Splice variant, non-pathogenic CNA |
| GENIE-DFCI-106199 | GENIE | GENIE-DFCI-106199-436842 | Metastasis | yes | yes | no | SNV | Q499Vfs*9, non-pathogenic CNA |
| GENIE-DFCI-123573 | GENIE | GENIE-DFCI-123573-1149676 | Primary | yes | yes | no | SNV | I605Yfs*9, non-pathogenic CNA |
| GENIE-DFCI-170500 | GENIE | GENIE-DFCI-170500-1525865 | Metastasis | yes | yes | no | SNV | E1550Afs*4, F1546Lfs*22, non-pathogenic CNA |
| GENIE-DUKE-P246 | GENIE | GENIE-DUKE-P246-S249 | Primary | yes | no | yes | SNV | E1456*, non-pathogenic CNA |
| GENIE-DUKE-P289 | GENIE | GENIE-DUKE-P289-S292 | Metastasis | yes | no | yes | SNV | H2093Tfs*26, non-pathogenic CNA |
| GENIE-DUKE-P433 | GENIE | GENIE-DUKE-P433-S436 | Metastasis | yes | no | yes | SNV | V1283Efs*10, non-pathogenic SNV |
| GENIE-DUKE-P7 | GENIE | GENIE-DUKE-P7-S7 | Primary | yes | no | yes | CNA | Deep Deletion |
| GENIE-GRCC-2bc9ccs2 | GENIE | GENIE-GRCC-2bc9ccs2-sample-a | Metastasis | yes | no | no | SNV | S1970* |
| GENIE-GRCC-fb2c482d | GENIE | GENIE-GRCC-fb2c482d-metastasis-a | Metastasis | yes | no | no | SNV | S871* |
| GENIE-GRCC-xqiohc18 | GENIE | GENIE-GRCC-xqiohc18-sample-a | Metastasis | yes | no | no | SNV | E49* |
| GENIE-MSK-P-0000441 | GENIE | GENIE-MSK-P-0000441-T01-IM3 | Metastasis | yes | yes | yes | SNV | L2362Cfs*5, non-pathogenic CNA |
| GENIE-MSK-P-0000541 | GENIE | GENIE-MSK-P-0000541-T03-IM6 | Metastasis | yes | yes | yes | SV | BRCA2-intragenic fusion, non-pathogenic CNA |
| GENIE-MSK-P-0000672 | GENIE | GENIE-MSK-P-0000672-T01-IM3 | Primary | yes | yes | yes | SNV | Y949Lfs*10, non-pathogenic CNA |
| GENIE-MSK-P-0000964 | GENIE | GENIE-MSK-P-0000964-T01-IM3 | Primary | yes | yes | yes | SNV | K2075*, non-pathogenic CNA |
| GENIE-MSK-P-0000964 | GENIE | GENIE-MSK-P-0000964-T03-IM5 | Metastasis | yes | yes | yes | SNV | K2075*, non-pathogenic CNA |
| GENIE-MSK-P-0001449 | GENIE | GENIE-MSK-P-0001449-T01-IM3 | Primary | yes | yes | yes | SNV | E2981Rfs*37, non-pathogenic CNA |
| GENIE-MSK-P-0001449 | GENIE | GENIE-MSK-P-0001449-T02-IM5 | Metastasis | yes | yes | yes | SNV | E2981Rfs*37, non-pathogenic CNA |
| GENIE-MSK-P-0001493 | GENIE | GENIE-MSK-P-0001493-T01-IM3 | Primary | yes | yes | yes | SNV | E897*, non-pathogenic CNA |
| GENIE-MSK-P-0001822 | GENIE | GENIE-MSK-P-0001822-T01-IM3 | Primary | yes | yes | yes | CNA | Deep Deletion |
| GENIE-MSK-P-0001829 | GENIE | GENIE-MSK-P-0001829-T02-IM5 | Metastasis | yes | yes | yes | SNV | T3033Nfs*11, non-pathogenic CNA |
| GENIE-MSK-P-0002398 | GENIE | GENIE-MSK-P-0002398-T01-IM3 | Metastasis | yes | yes | yes | CNA | Deep Deletion |
| GENIE-MSK-P-0002398 | GENIE | GENIE-MSK-P-0002398-T02-IM3 | Primary | yes | yes | yes | CNA | Deep Deletion |
| GENIE-MSK-P-0002516 | GENIE | GENIE-MSK-P-0002516-T01-IM3 | Metastasis | yes | yes | yes | CNA | Deep Deletion |
| GENIE-MSK-P-0002962 | GENIE | GENIE-MSK-P-0002962-T01-IM3 | Primary | yes | yes | yes | CNA | Deep Deletion |
| GENIE-MSK-P-0003468 | GENIE | GENIE-MSK-P-0003468-T01-IM5 | Metastasis | yes | yes | yes | SNV/CNA | K1860*, Deep Deletion |
| GENIE-MSK-P-0003852 | GENIE | GENIE-MSK-P-0003852-T01-IM5 | Primary | yes | yes | yes | CNA | Deep Deletion |
| GENIE-MSK-P-0004063 | GENIE | GENIE-MSK-P-0004063-T01-IM5 | Primary | yes | yes | yes | SNV | N1055Ifs*5, non-pathogenic CNA |
| GENIE-MSK-P-0004489 | GENIE | GENIE-MSK-P-0004489-T01-IM5 | Metastasis | yes | yes | yes | CNA | Deep Deletion |
| GENIE-MSK-P-0004593 | GENIE | GENIE-MSK-P-0004593-T01-IM5 | Metastasis | yes | yes | yes | CNA | Deep Deletion |
| GENIE-MSK-P-0004735 | GENIE | GENIE-MSK-P-0004735-T01-IM5 | Primary | yes | yes | yes | SNV | R645Efs*15, non-pathogenic CNA |
| GENIE-MSK-P-0004735 | GENIE | GENIE-MSK-P-0004735-T02-IM6 | Metastasis | yes | yes | yes | SNV | R645Efs*15 pathogenic, S1982_G1983delinsRE non-pathogenic |
| GENIE-MSK-P-0004892 | GENIE | GENIE-MSK-P-0004892-T01-IM5 | Metastasis | yes | yes | yes | SV | INSC-BRCA2 fusion, non-pathogenic CNA |
| GENIE-MSK-P-0004946 | GENIE | GENIE-MSK-P-0004946-T01-IM5 | Primary | yes | yes | yes | SV | BRCA2-intragenic fusion, non-pathogenic CNA |
| GENIE-MSK-P-0005774 | GENIE | GENIE-MSK-P-0005774-T01-IM5 | Primary | yes | yes | yes | SNV | E532Rfs*3, non-pathogenic CNA |
| GENIE-MSK-P-0006461 | GENIE | GENIE-MSK-P-0006461-T01-IM5 | Metastasis | yes | yes | yes | SNV | E2198Nfs*4, non-pathogenic CNA |
| GENIE-MSK-P-0006749 | GENIE | GENIE-MSK-P-0006749-T01-IM5 | Metastasis | yes | yes | yes | SNV | X228_splice, non-pathogenic CNA |
| GENIE-MSK-P-0006994 | GENIE | GENIE-MSK-P-0006994-T01-IM5 | Metastasis | yes | yes | yes | CNA | Deep Deletion |
| GENIE-MSK-P-0007289 | GENIE | GENIE-MSK-P-0007289-T02-IM5 | Metastasis | yes | yes | yes | CNA | Deep Deletion |
| GENIE-MSK-P-0007366 | GENIE | GENIE-MSK-P-0007366-T01-IM5 | Primary | yes | yes | yes | SNV | D1054Efs*9, non-pathogenic CNA |
| GENIE-MSK-P-0008098 | GENIE | GENIE-MSK-P-0008098-T01-IM5 | Metastasis | yes | yes | yes | CNA | Deep Deletion |
| GENIE-MSK-P-0008769 | GENIE | GENIE-MSK-P-0008769-T02-IM5 | Metastasis | yes | yes | yes | SNV | X3086_splice, non-pathogenic CNA |
| GENIE-MSK-P-0009399 | GENIE | GENIE-MSK-P-0009399-T01-IM5 | Metastasis | yes | yes | yes | CNA | Deep Deletion |
| GENIE-MSK-P-0009546 | GENIE | GENIE-MSK-P-0009546-T01-IM5 | Primary | yes | yes | yes | SNV/CNA | G1122Efs*28, Deep Deletion |
| GENIE-MSK-P-0009654 | GENIE | GENIE-MSK-P-0009654-T01-IM5 | Metastasis | yes | yes | yes | SNV | P655Qfs*5, non-pathogenic CNA |
| GENIE-MSK-P-0009853 | GENIE | GENIE-MSK-P-0009853-T01-IM5 | Metastasis | yes | yes | yes | CNA | Deep Deletion |
| GENIE-MSK-P-0010382 | GENIE | GENIE-MSK-P-0010382-T01-IM5 | Metastasis | yes | yes | yes | CNA | Deep Deletion |
| GENIE-MSK-P-0010529 | GENIE | GENIE-MSK-P-0010529-T01-IM5 | Metastasis | yes | yes | yes | CNA | Deep Deletion |
| GENIE-MSK-P-0011363 | GENIE | GENIE-MSK-P-0011363-T01-IM5 | Primary | yes | yes | yes | SNV | H543Tfs*17, non-pathogenic CNA |
| GENIE-MSK-P-0011529 | GENIE | GENIE-MSK-P-0011529-T01-IM5 | Primary | yes | yes | yes | SNV | E1581*, non-pathogenic CNA |
| GENIE-MSK-P-0012046 | GENIE | GENIE-MSK-P-0012046-T01-IM5 | Primary | yes | yes | yes | CNA | Deep Deletion |
| GENIE-MSK-P-0012132 | GENIE | GENIE-MSK-P-0012132-T02-IM6 | Metastasis | yes | yes | yes | CNA | Deep Deletion |
| GENIE-MSK-P-0012145 | GENIE | GENIE-MSK-P-0012145-T01-IM5 | Primary | yes | yes | yes | SNV | S1099*, non-pathogenic CNA |
| GENIE-MSK-P-0012630 | GENIE | GENIE-MSK-P-0012630-T02-IM6 | Primary | yes | yes | yes | CNA | Deep Deletion |
| GENIE-MSK-P-0013729 | GENIE | GENIE-MSK-P-0013729-T01-IM5 | Metastasis | yes | yes | yes | CNA | Deep Deletion |
| GENIE-MSK-P-0013837 | GENIE | GENIE-MSK-P-0013837-T01-IM5 | Primary | yes | yes | yes | SNV | S2533Lfs*18, non-pathogenic CNA |
| GENIE-MSK-P-0014408 | GENIE | GENIE-MSK-P-0014408-T01-IM6 | Metastasis | yes | yes | yes | CNA | Deep Deletion |
| GENIE-MSK-P-0015570 | GENIE | GENIE-MSK-P-0015570-T01-IM6 | Primary | yes | yes | yes | SNV | I605Yfs*9, non-pathogenic CNA |
| GENIE-MSK-P-0015628 | GENIE | GENIE-MSK-P-0015628-T01-IM6 | Metastasis | yes | yes | yes | CNA | Deep Deletion |
| GENIE-MSK-P-0016296 | GENIE | GENIE-MSK-P-0016296-T01-IM6 | Metastasis | yes | yes | yes | SNV | E2846*, non-pathogenic CNA |
| GENIE-MSK-P-0017168 | GENIE | GENIE-MSK-P-0017168-T01-IM6 | Primary | yes | yes | yes | SNV | P2762Sfs*2, non-pathogenic CNA |
| GENIE-MSK-P-0017169 | GENIE | GENIE-MSK-P-0017169-T01-IM6 | Metastasis | yes | yes | yes | CNA | Deep Deletion |
| GENIE-MSK-P-0017524 | GENIE | GENIE-MSK-P-0017524-T01-IM6 | Metastasis | yes | yes | yes | SNV | P655Qfs*5, non-pathogenic CNA |
| GENIE-MSK-P-0019114 | GENIE | GENIE-MSK-P-0019114-T01-IM6 | Metastasis | yes | yes | yes | CNA | Deep Deletion |
| GENIE-MSK-P-0019199 | GENIE | GENIE-MSK-P-0019199-T02-IM6 | Metastasis | yes | yes | yes | SNV | T3033Lfs*29 |
| GENIE-MSK-P-0019782 | GENIE | GENIE-MSK-P-0019782-T01-IM6 | Primary | yes | yes | yes | SNV | X2479_splice, non-pathogenic CNA |
| GENIE-MSK-P-0019930 | GENIE | GENIE-MSK-P-0019930-T01-IM6 | Metastasis | yes | yes | yes | CNA | Deep Deletion |
| GENIE-MSK-P-0019937 | GENIE | GENIE-MSK-P-0019937-T01-IM6 | Metastasis | yes | yes | yes | SV | NBEA-BRCA2 fusion, non-pathogenic CNA |
| GENIE-MSK-P-0019975 | GENIE | GENIE-MSK-P-0019975-T01-IM6 | Primary | yes | yes | yes | SNV | V1804Kfs*2, non-pathogenic CNA |
| GENIE-MSK-P-0021254 | GENIE | GENIE-MSK-P-0021254-T01-IM6 | Primary | yes | yes | yes | SNV | N1316Kfs*3, non-pathogenic CNA |
| GENIE-MSK-P-0021922 | GENIE | GENIE-MSK-P-0021922-T01-IM6 | Metastasis | yes | yes | yes | CNA | Deep Deletion |
| GENIE-MSK-P-0022114 | GENIE | GENIE-MSK-P-0022114-T01-IM6 | Metastasis | yes | yes | yes | CNA | Deep Deletion |
| GENIE-MSK-P-0022114 | GENIE | GENIE-MSK-P-0022114-T02-IM6 | Primary | yes | yes | yes | CNA | Deep Deletion |
| GENIE-MSK-P-0022249 | GENIE | GENIE-MSK-P-0022249-T01-IM6 | Primary | yes | yes | yes | CNA | Deep Deletion |
| GENIE-MSK-P-0022383 | GENIE | GENIE-MSK-P-0022383-T01-IM6 | Primary | yes | yes | yes | SV | NUFIP1-BRCA2 fusion, non-pathogenic CNA |
| GENIE-MSK-P-0022707 | GENIE | GENIE-MSK-P-0022707-T01-IM6 | Metastasis | yes | yes | yes | SNV | N1784Kfs*3, non-pathogenic CNA |
| GENIE-MSK-P-0022782 | GENIE | GENIE-MSK-P-0022782-T01-IM6 | Metastasis | yes | yes | yes | CNA | Deep Deletion |
| GENIE-MSK-P-0023419 | GENIE | GENIE-MSK-P-0023419-T01-IM6 | Metastasis | yes | yes | yes | CNA | Deep Deletion |
| GENIE-MSK-P-0023561 | GENIE | GENIE-MSK-P-0023561-T01-IM6 | Metastasis | yes | yes | yes | CNA | Deep Deletion |
| GENIE-MSK-P-0023855 | GENIE | GENIE-MSK-P-0023855-T01-IM6 | Primary | yes | yes | yes | SNV | W1692Mfs*3, non-pathogenic CNA |
| GENIE-MSK-P-0023930 | GENIE | GENIE-MSK-P-0023930-T01-IM6 | Primary | yes | yes | yes | SV | KL-BRCA2 fusion, non-pathogenic CNA |
| GENIE-MSK-P-0023961 | GENIE | GENIE-MSK-P-0023961-T01-IM6 | Metastasis | yes | yes | yes | CNA | Deep Deletion |
| GENIE-MSK-P-0024660 | GENIE | GENIE-MSK-P-0024660-T01-IM6 | Metastasis | yes | yes | yes | SNV | I2948Lfs*28, non-pathogenic CNA |
| GENIE-MSK-P-0025097 | GENIE | GENIE-MSK-P-0025097-T01-IM6 | Metastasis | yes | yes | yes | SNV | L1227Qfs*5, non-pathogenic CNA |
| GENIE-MSK-P-0025243 | GENIE | GENIE-MSK-P-0025243-T01-IM6 | Primary | yes | yes | yes | SNV | Q2164*, non-pathogenic CNA |
| GENIE-MSK-P-0025276 | GENIE | GENIE-MSK-P-0025276-T01-IM6 | Metastasis | yes | yes | yes | SNV | S1262*, non-pathogenic CNA |
| GENIE-MSK-P-0025550 | GENIE | GENIE-MSK-P-0025550-T01-IM6 | Primary | yes | yes | yes | CNA | Deep Deletion |
| GENIE-MSK-P-0026768 | GENIE | GENIE-MSK-P-0026768-T01-IM6 | Metastasis | yes | yes | yes | CNA | Deep Deletion |
| GENIE-MSK-P-0026871 | GENIE | GENIE-MSK-P-0026871-T01-IM6 | Primary | yes | yes | yes | SNV | S1064*, non-pathogenic CNA |
| GENIE-MSK-P-0028587 | GENIE | GENIE-MSK-P-0028587-T01-IM6 | Primary | yes | yes | yes | SNV | S552Ffs*8, non-pathogenic CNA |
| GENIE-MSK-P-0029336 | GENIE | GENIE-MSK-P-0029336-T01-IM6 | Metastasis | yes | yes | yes | SV | LINC00371-BRCA2 fusion, non-pathogenic CNA |
| GENIE-MSK-P-0029339 | GENIE | GENIE-MSK-P-0029339-T01-IM6 | Metastasis | yes | yes | yes | CNA | Deep Deletion |
| GENIE-MSK-P-0030831 | GENIE | GENIE-MSK-P-0030831-T01-IM6 | Metastasis | yes | yes | yes | SNV | N2145Ifs*23, non-pathogenic CNA |
| GENIE-MSK-P-0031419 | GENIE | GENIE-MSK-P-0031419-T01-IM6 | Metastasis | yes | yes | yes | CNA | Deep Deletion |
| GENIE-MSK-P-0031712 | GENIE | GENIE-MSK-P-0031712-T01-IM6 | Metastasis | yes | yes | yes | CNA | Deep Deletion |
| GENIE-MSK-P-0032480 | GENIE | GENIE-MSK-P-0032480-T01-IM6 | Primary | yes | yes | yes | SNV | K2940*, non-pathogenic CNA |
| GENIE-MSK-P-0032757 | GENIE | GENIE-MSK-P-0032757-T01-IM6 | Metastasis | yes | yes | yes | CNA | Deep Deletion |
| GENIE-MSK-P-0033660 | GENIE | GENIE-MSK-P-0033660-T01-IM6 | Metastasis | yes | yes | yes | CNA | Deep Deletion |
| GENIE-MSK-P-0033701 | GENIE | GENIE-MSK-P-0033701-T02-IM6 | Metastasis | yes | yes | yes | SNV | T1974_S1982delinsL, non-pathogenic CNA |
| GENIE-MSK-P-0034300 | GENIE | GENIE-MSK-P-0034300-T01-IM6 | Metastasis | yes | yes | yes | SNV | E1158*, non-pathogenic CNA |
| GENIE-MSK-P-0034632 | GENIE | GENIE-MSK-P-0034632-T01-IM6 | Metastasis | yes | yes | yes | SNV | E732*, non-pathogenic CNA |
| GENIE-MSK-P-0034632 | GENIE | GENIE-MSK-P-0034632-T02-IM6 | Primary | yes | yes | yes | SNV | E732*, non-pathogenic CNA |
| GENIE-MSK-P-0034788 | GENIE | GENIE-MSK-P-0034788-T01-IM6 | Primary | yes | yes | yes | CNA | Deep Deletion |
| GENIE-MSK-P-0035133 | GENIE | GENIE-MSK-P-0035133-T01-IM6 | Primary | yes | yes | yes | SNV | X2479_splice pathogenic, H415Y non-pathogenic, I1470M non-pathogenic, non-pathogenic CNA |
| GENIE-MSK-P-0035746 | GENIE | GENIE-MSK-P-0035746-T01-IM6 | Metastasis | yes | yes | yes | SNV | K1018*, non-pathogenic CNA |
| GENIE-MSK-P-0035765 | GENIE | GENIE-MSK-P-0035765-T01-IM6 | Metastasis | yes | yes | yes | SV/CNA | BRCA2-VCAN Fusion, Deep Deletion |
| GENIE-MSK-P-0036572 | GENIE | GENIE-MSK-P-0036572-T01-IM6 | Primary | yes | yes | yes | SNV | I2330Hfs*3, non-pathogenic CNA |
| GENIE-MSK-P-0037151 | GENIE | GENIE-MSK-P-0037151-T01-IM6 | Primary | yes | yes | yes | SNV | X1831_splice, non-pathogenic CNA |
| GENIE-MSK-P-0037423 | GENIE | GENIE-MSK-P-0037423-T01-IM6 | Primary | yes | yes | yes | SNV | H179Nfs*7, non-pathogenic CNA |
| GENIE-MSK-P-0037751 | GENIE | GENIE-MSK-P-0037751-T01-IM6 | Metastasis | yes | yes | yes | CNA | Deep Deletion |
| GENIE-MSK-P-0037801 | GENIE | GENIE-MSK-P-0037801-T01-IM6 | Metastasis | yes | yes | yes | CNA | Deep Deletion |
| GENIE-MSK-P-0038412 | GENIE | GENIE-MSK-P-0038412-T01-IM6 | Primary | yes | yes | yes | SNV | W1692Mfs*3, non-pathogenic CNA |
| GENIE-MSK-P-0039753 | GENIE | GENIE-MSK-P-0039753-T01-IM6 | Primary | yes | yes | yes | SNV | Y2905*, non-pathogenic CNA |
| GENIE-MSK-P-0040458 | GENIE | GENIE-MSK-P-0040458-T01-IM6 | Primary | yes | yes | yes | SV/SNV | X2918_splice, BRCA2 intragenic fusion, non-pathogenic CNA |
| GENIE-MSK-P-0040739 | GENIE | GENIE-MSK-P-0040739-T01-IM6 | Metastasis | yes | yes | yes | SNV | K589Vfs*7, non-pathogenic CNA |
| GENIE-MSK-P-0041027 | GENIE | GENIE-MSK-P-0041027-T01-IM6 | Metastasis | yes | yes | yes | CNA | Deep Deletion |
| GENIE-MSK-P-0041610 | GENIE | GENIE-MSK-P-0041610-T01-IM6 | Primary | yes | yes | yes | CNA | Deep Deletion |
| GENIE-MSK-P-0042144 | GENIE | GENIE-MSK-P-0042144-T01-IM6 | Primary | yes | yes | yes | SNV | T3085Nfs*26, N1198Kfs*2, non-pathogenic CNA |
| GENIE-MSK-P-0042332 | GENIE | GENIE-MSK-P-0042332-T01-IM6 | Metastasis | yes | yes | yes | SV/SNV | L2647*, BRCA2 intragenic fusion, non-pathogenic CNA |
| GENIE-MSK-P-0042990 | GENIE | GENIE-MSK-P-0042990-T01-IM6 | Primary | yes | yes | yes | CNA | Deep Deletion |
| GENIE-MSK-P-0043565 | GENIE | GENIE-MSK-P-0043565-T01-IM6 | Metastasis | yes | yes | yes | CNA | Deep Deletion |
| GENIE-UHN-OCT114720 | GENIE | GENIE-UHN-OCT114720-ARC1 | Metastasis | yes | no | no | SNV | D252Vfs*24 |
| GENIE-UHN-OCT549985 | GENIE | GENIE-UHN-OCT549985-ARC1 | Primary | yes | no | no | SNV | L2357Vfs*2 |
| GENIE-VICC-314976 | GENIE | GENIE-VICC-314976-unk-1 | Primary | yes | yes | yes | SNV | P606Tfs*10 pathogenic, G522R unknown effect, non-pathogenic CNA |
| GENIE-VICC-531433 | GENIE | GENIE-VICC-531433-unk-1 | Metastasis | yes | yes | yes | SNV | E2558Vfs*7, non-pathogenic CNA |
| GENIE-VICC-534440 | GENIE | GENIE-VICC-534440-unk-1 | Metastasis | yes | yes | yes | SNV | M124Ifs*11, non-pathogenic CNA |
| GENIE-VICC-745559 | GENIE | GENIE-VICC-745559-unk-1 | Metastasis | yes | yes | yes | SNV | E2769*, non-pathogenic CNA |
| GENIE-YALE-TPL360 | GENIE | GENIE-YALE-TPL360-1 | Primary | yes | yes | no | SNV | E350* |
| GENIE-YALE-TPL628 | GENIE | GENIE-YALE-TPL628-1 | Primary | yes | yes | no | SNV | D1386Ifs*2 |
| TCGA-CH-5748 | TCGA | TCGA-CH-5748-01 | Primary | yes | yes | yes | CNA | Deep Deletion |
| TCGA-CH-5754 | TCGA | TCGA-CH-5754-01 | Primary | yes | yes | yes | SNV | N433Tfs*27 |
| TCGA-EJ-5495 | TCGA | TCGA-EJ-5495-01 | Primary | yes | yes | yes | SNV | V726Sfs*25, non-pathogenic CNA |
| TCGA-EJ-5527 | TCGA | TCGA-EJ-5527-01 | Primary | yes | yes | yes | CNA | Deep Deletion |
| TCGA-EJ-5531 | TCGA | TCGA-EJ-5531-01 | Primary | yes | yes | no | CNA | Deep Deletion |
| TCGA-EJ-7328 | TCGA | TCGA-EJ-7328-01 | Primary | yes | yes | yes | SNV | S3364Ifs*4 |
| TCGA-EJ-A65F | TCGA | TCGA-EJ-A65F-01 | Primary | yes | yes | yes | CNA | Deep Deletion |
| TCGA-EJ-A65M | TCGA | TCGA-EJ-A65M-01 | Primary | yes | yes | yes | CNA | Deep Deletion |
| TCGA-HC-7210 | TCGA | TCGA-HC-7210-01 | Primary | yes | yes | yes | CNA | Deep Deletion |
| TCGA-HC-7738 | TCGA | TCGA-HC-7738-01 | Primary | yes | yes | yes | CNA | Deep Deletion |
| TCGA-J4-AAU2 | TCGA | TCGA-J4-AAU2-01 | Primary | yes | yes | no | CNA | Deep Deletion |
| TCGA-J9-A8CM | TCGA | TCGA-J9-A8CM-01 | Primary | yes | yes | yes | SNV | C916Vfs*2 |
| TCGA-KC-A7F3 | TCGA | TCGA-KC-A7F3-01 | Primary | yes | yes | yes | CNA | Deep Deletion |
| TCGA-KK-A8IB | TCGA | TCGA-KK-A8IB-01 | Primary | yes | yes | yes | CNA | Deep Deletion |
| TCGA-V1-A9OH | TCGA | TCGA-V1-A9OH-01 | Primary | yes | yes | no | SNV | N213Mfs*17 |
| TCGA-V1-A9OX | TCGA | TCGA-V1-A9OX-01 | Primary | yes | yes | no | CNA | Deep Deletion |
| TCGA-V1-A9OY | TCGA | TCGA-V1-A9OY-01 | Primary | yes | yes | yes | CNA | Deep Deletion |
| TCGA-V1-A9Z9 | TCGA | TCGA-V1-A9Z9-01 | Primary | yes | yes | no | CNA | Deep Deletion |
| TCGA-VN-A88R | TCGA | TCGA-VN-A88R-01 | Primary | yes | yes | yes | CNA | Deep Deletion |
| TCGA-XQ-A8TA | TCGA | TCGA-XQ-A8TA-01 | Primary | yes | yes | yes | CNA | Deep Deletion |
| TCGA-YL-A8HO | TCGA | TCGA-YL-A8HO-01 | Primary | yes | yes | yes | CNA | Deep Deletion |
| TCGA-ZG-A9NI | TCGA | TCGA-ZG-A9NI-01 | Primary | yes | yes | yes | CNA | Deep Deletion |

**Supplementary Table 2**. List of candidate genes identified a priori and included in analyses due to high alteration frequency

| **Hereditary Cancer Genes** | **BRCA2 Interactor Genes** | **Commonly Altered Prostate Cancer Genes** |
| --- | --- | --- |
| ATM | ABRA1 | APC |
| ATR | BACH1 | AR |
| BRCA1 | BARD1 | BRAF |
| BRIP1 | BCCIP | CDK12 |
| CHEK2 | BRCC36 | CDKN1B |
| EPCAM | BRE | CHD1 |
| FANCA | CTIP | CTNNB1 |
| GEN1 | EMSY | DNAPK |
| HOXB13 | MERIT40 | ERF |
| MLH1 | MRE11 | ERG |
| MSH2 | MRE11A | ETV1 |
| MSH6 | NBS1 | ETV4 |
| NBN | POU5F1P1 | FOXA1 |
| PALB2 | RAD50 | IDH1 |
| PMS2 | RAD51 | KMT2C |
| RAD51C | RAD51AP1 | KMT2D |
| RAD51D | RAD51B | MED12 |
| TP53 | RAP80 | MYC |
|  | RASSF1A | NCOR1 |
|  | TOPBP1 | NCOR2 |
|  | ZNF350 | PIK3CA |
|  |  | PTEN |
|  |  | RB1 |
|  |  | RNF43 |
|  |  | SPOP |
|  |  | TMPRSS2 |
|  |  | ZFHX3 |
|  |  | ZMYM3 |

| **Additional Top Genes**  **Primary SNV** | **Additional Top Genes**  **Metastatic SNV** | **Additional Top Genes**  **Primary CNA** | **Additional Top Genes**  **Metastatic CNA** |
| --- | --- | --- | --- |
| CACNA1E | ASXL1 | AK9 | AGO2 |
| CSMD1 | GRIN2A | CNBD1 | AMER1 |
| CSMD3 | JAK1 | CPQ | CYSLTR2 |
| FAT3 | KDM6A | CSMD3 | DUSP4 |
| KDM6A | MGA | DCAF13 | FGFR1 |
| LRP1B | SPEN | FOXO3 | LYN |
| SPEN |  | KCNB2 | PRDM14 |
| SPTA1 |  | KLLN | PREX2 |
| SYNE1 |  | LINC00535 | RAD21 |
|  |  | MAP3K7 | RECQL4 |
|  |  | NEK3 | TCEB1 |
|  |  | NSMCE2 | WHSC1L1 |
|  |  | RAD54B |  |
|  |  | RSPO2 |  |
|  |  | RUNX1T1 |  |
|  |  | SNX31 |  |
|  |  | TPD52 |  |
|  |  | UBR5 |  |
|  |  | WISP3 |  |

**Supplementary Table 3**. Clinical and pathological characteristics of primary and metastatic tumors by tumor *BRCA2* status.

|  | **Primary** | | **Metastatic** | |
| --- | --- | --- | --- | --- |
|  | **BRCA2^d^ (n=138)** | **BRCA2^i^ (n=2398)** | **BRCA2^d^ (n=85)** | **BRCA2^i^ (n=814)** |
| **Data Source, n (%)** |  |  |  |  |
| GENIE | 68 (49.3) | 1443 (60.2) | 81 (95.3) | 808 (99.3) |
| ICGC | 48 (34.8) | 483 (20.1) | 4 (4.7) | 6 (0.7) |
| TCGA | 22 (15.9) | 472 (19.7) | 0 (0.0) | 0 (0.0) |
| **Age at Diagnosis (ICGC, TCGA), mean (SD)** | 64.4 (6.5) | 62.2 (7.3) | 60.5 (4.4) | 61.5 (7.6) |
| n missing | 0 | 13 | 0 | 0 |
| **Age at Sequencing (GENIE), mean (SD)** | 64.3 (8.5) | 65.5 (8.6) | 66.6 (9.2) | 68.4 (9.0) |
| n missing | 0 | 3 | 0 | 2 |
| **Race, n (%)** |  |  |  |  |
| White | 64 (46.4) | 1305 (54.4) | 63 (74.1) | 639 (78.5) |
| Black | 5 (3.6) | 139 (5.8) | 3 (3.5) | 66 (8.1) |
| Asian | 0 (0.0) | 44 (1.8) | 3 (3.5) | 32 (3.9) |
| Other | 1 (0.1) | 24 (1.0) | 2 (2.4) | 14 (1.7) |
| Unknown | 68 (49.3) | 886 (36.9) | 14 (16.5) | 63 (7.7) |
| **Ethnicity, n (%)** |  |  |  |  |
| Hispanic | 1 (0.7) | 55 (2.3) | 4 (4.7) | 33 (4.1) |
| non-Hispanic | 69 (50.0) | 1422 (59.3) | 68 (80.0) | 705 (86.6) |
| Unknown | 68 (49.3) | 921 (38.4) | 13 (15.3) | 76 (9.3) |
| **Tumor T Stage, n (%)** |  |  |  |  |
| T1-T2a | 41 (29.7) | 323 (13.5) | 0 (0.0) | 1 (0.1) |
| T2b-T2c | 14 (10.1) | 285 (11.9) | 1 (1.2) | 1 (0.1) |
| T3-T4 | 14 (10.1) | 330 (13.8) | 3 (3.5) | 3 (0.4) |
| Unknown | 69 (50.0) | 1460 (60.9) | 81 (95.3) | 809 (99.4) |
| **Tumor Gleason Grade, n (%)** |  |  |  |  |
| 6 | 7 (5.1) | 58 (2.4) | 0 (0.0) | 0 (0.0) |
| 7 (3+4) | 16 (11.6) | 160 (6.7) | 0 (0.0) | 0 (0.0) |
| 7 (4+3) | 8 (5.8) | 72 (3.0) | 0 (0.0) | 0 (0.0) |
| 8 | 3 (2.2) | 19 (0.8) | 0 (0.0) | 0 (0.0) |
| 9-10 | 2 (1.4) | 12 (0.5) | 0 (0.0) | 0 (0.0) |
| Unknown | 102 (73.9) | 2077 (86.6) | 85 (100.0) | 814 (100.0) |

BRCA2^d^ = BRCA2-deficient; BRCA2^i^ = BRCA2-intact

**Supplementary Table 4**. Pathogenic SNV frequency by BRCA2 status in primary tumors

| **Gene** | **SNVs**  **BRCA2^d^ (n)** | **SNV Freq.**  **BRCA2^d^ (%)** | **SNVs**  **BRCA2^i^ (n)** | **SNV Freq.**  **BRCA2^i^ (%)** | **Nominal**  **P-Value** | **Significant** |
| --- | --- | --- | --- | --- | --- | --- |
| KMT2D | 17 | 12.5 | 111 | 4.6 | 0.00041 | yes |
| CSMD1 | 4 | 5.7 | 19 | 2 | 0.03319 | no |
| MYC | 3 | 2.2 | 12 | 0.5 | 0.04437 | no |
| BACH1 | 1 | 1.4 | 0 | 0 | 0.05444 | no |
| PMS2 | 2 | 1.4 | 7 | 0.3 | 0.08238 | no |
| MSH6 | 3 | 2.2 | 17 | 0.7 | 0.09136 | no |
| RAD51 | 1 | 0.8 | 1 | 0 | 0.10593 | no |
| GEN1 | 1 | 1.2 | 1 | 0.1 | 0.10593 | no |
| RNF43 | 4 | 3.1 | 30 | 1.3 | 0.11024 | no |
| MLH1 | 2 | 1.4 | 9 | 0.4 | 0.1173 | no |
| BARD1 | 2 | 1.6 | 9 | 0.4 | 0.1173 | no |
| SYNE1 | 4 | 5.7 | 31 | 3.2 | 0.11948 | no |
| ATR | 3 | 2.3 | 20 | 0.9 | 0.12634 | no |
| CSMD3 | 4 | 5.7 | 32 | 3.4 | 0.12904 | no |
| CACNA1E | 3 | 4.3 | 21 | 2.2 | 0.13893 | no |
| EPCAM | 1 | 0.8 | 2 | 0.1 | 0.15464 | no |
| FANCA | 2 | 1.4 | 11 | 0.5 | 0.15514 | no |
| BRCA1 | 2 | 1.4 | 13 | 0.5 | 0.19492 | no |
| SPEN | 5 | 4.1 | 48 | 2.1 | 0.20854 | no |
| MRE11A | 1 | 0.8 | 4 | 0.2 | 0.2443 | no |
| FOXA1 | 7 | 5.6 | 195 | 8.7 | 0.25614 | no |
| IDH1 | 2 | 1.4 | 16 | 0.7 | 0.25643 | no |
| TP53 | 16 | 11.6 | 371 | 15.5 | 0.27244 | no |
| BRAF | 4 | 2.9 | 36 | 1.5 | 0.27493 | no |
| TMPRSS2 | 2 | 1.5 | 17 | 0.8 | 0.2771 | no |
| PALB2 | 2 | 1.4 | 17 | 0.7 | 0.2771 | no |
| SPOP | 9 | 6.9 | 232 | 10.3 | 0.29422 | no |
| NBN | 1 | 0.7 | 6 | 0.3 | 0.3245 | no |
| MED12 | 4 | 3.1 | 44 | 2 | 0.32977 | no |
| PTEN | 10 | 7.2 | 129 | 5.4 | 0.33498 | no |
| CHEK2 | 1 | 0.7 | 8 | 0.3 | 0.39625 | no |
| CDKN1B | 0 | 0 | 26 | 1.1 | 0.39689 | no |
| RAD50 | 1 | 0.8 | 9 | 0.4 | 0.42924 | no |
| NCOR2 | 1 | 1.4 | 9 | 0.9 | 0.42924 | no |
| CDK12 | 6 | 4.6 | 78 | 3.5 | 0.45925 | no |
| APC | 6 | 4.4 | 79 | 3.3 | 0.46333 | no |
| NCOR1 | 3 | 2.5 | 36 | 1.6 | 0.46841 | no |
| HOXB13 | 1 | 0.8 | 11 | 0.5 | 0.48993 | no |
| ETV1 | 1 | 0.7 | 11 | 0.5 | 0.48993 | no |
| ERG | 1 | 0.8 | 11 | 0.5 | 0.48993 | no |
| BRIP1 | 1 | 0.7 | 15 | 0.6 | 0.59276 | no |
| ZMYM3 | 0 | 0 | 18 | 1.9 | 0.62027 | no |
| LRP1B | 2 | 2.7 | 33 | 3.4 | 0.71574 | no |
| ERF | 1 | 1 | 35 | 1.9 | 0.71961 | no |
| CTNNB1 | 3 | 2.2 | 43 | 1.8 | 0.73704 | no |
| KDM6A | 4 | 2.9 | 66 | 2.8 | 0.79061 | no |
| PIK3CA | 4 | 2.9 | 67 | 2.8 | 0.79334 | no |
| ATM | 5 | 3.6 | 77 | 3.2 | 0.80217 | no |
| KMT2C | 7 | 5.8 | 111 | 4.9 | 0.83407 | no |
| RAD51B | 0 | 0 | 7 | 0.3 | 1 | no |
| RAD51C | 0 | 0 | 1 | 0 | 1 | no |
| RAD51D | 0 | 0 | 1 | 0 | 1 | no |
| MSH2 | 0 | 0 | 16 | 0.7 | 1 | no |
| FAT3 | 1 | 1.4 | 21 | 2.2 | 1 | no |
| CHD1 | 0 | 0 | 9 | 0.9 | 1 | no |
| BRE | 0 | 0 | 1 | 0.1 | 1 | no |
| SPTA1 | 1 | 1.4 | 26 | 2.7 | 1 | no |
| BCCIP | 0 | 0 | 1 | 0.1 | 1 | no |
| ZFHX3 | 4 | 3.6 | 77 | 3.6 | 1 | no |
| TOPBP1 | 0 | 0 | 2 | 0.2 | 1 | no |
| AR | 1 | 0.7 | 17 | 0.7 | 1 | no |
| ETV4 | 0 | 0 | 2 | 0.2 | 1 | no |
| RB1 | 1 | 0.7 | 27 | 1.1 | 1 | no |
| ABRA1 | 0 | 0 | 0 | 0 | N/A | no |
| BRCC36 | 0 | 0 | 0 | 0 | N/A | no |
| CTIP | 0 | 0 | 0 | 0 | N/A | no |
| DNAPK | 0 | 0 | 0 | 0 | N/A | no |
| EMSY | 0 | 0 | 0 | 0 | N/A | no |
| MERIT40 | 0 | 0 | 0 | 0 | N/A | no |
| MRE11 | 0 | 0 | 0 | 0 | N/A | no |
| NBS1 | 0 | 0 | 0 | 0 | N/A | no |
| POU5F1P1 | 0 | 0 | 0 | 0 | N/A | no |
| RAD51AP1 | 0 | 0 | 0 | 0 | N/A | no |
| RAP80 | 0 | 0 | 0 | 0 | N/A | no |
| RASSF1A | 0 | 0 | 0 | 0 | N/A | no |
| ZNF350 | 0 | 0 | 0 | 0 | N/A | no |

BRCA2^d^ = BRCA2-deficient; BRCA2^i^ = BRCA2-intact

**Supplementary Table 5**. Total SNV frequency by BRCA2 status in primary tumors

| **Gene** | **SNVs**  **BRCA2^d^ (n)** | **SNV Freq.**  **BRCA2^d^ (%)** | **SNVs**  **BRCA2^i^ (n)** | **SNV Freq.**  **BRCA2^i^ (%)** | **Nominal**  **P-Value** | **Significant** |
| --- | --- | --- | --- | --- | --- | --- |
| CSMD3 | 47 | 67.1 | 423 | 44.3 | 1.00E-05 | yes |
| LRP1B | 49 | 66.2 | 448 | 46.2 | 1.00E-05 | yes |
| CSMD1 | 45 | 64.3 | 450 | 47.2 | 0.00016 | yes |
| SYNE1 | 27 | 38.6 | 223 | 23.4 | 0.00033 | yes |
| FANCA | 8 | 5.8 | 31 | 1.3 | 0.00092 | yes |
| BARD1 | 8 | 6.2 | 35 | 1.6 | 0.0018 | yes |
| ERG | 19 | 14.3 | 154 | 6.8 | 0.00256 | yes |
| NCOR2 | 13 | 18.6 | 88 | 9.2 | 0.00271 | yes |
| MYC | 5 | 3.6 | 14 | 0.6 | 0.00278 | yes |
| KMT2D | 18 | 13.2 | 140 | 5.8 | 0.00293 | yes |
| BACH1 | 16 | 21.9 | 125 | 12.9 | 0.00366 | yes |
| CACNA1E | 22 | 31.4 | 214 | 22.4 | 0.00974 | no |
| ETV1 | 10 | 7.4 | 70 | 2.9 | 0.01025 | no |
| NBN | 6 | 4.3 | 30 | 1.3 | 0.01174 | no |
| BRIP1 | 10 | 7.4 | 72 | 3 | 0.01213 | no |
| MLH1 | 5 | 3.6 | 22 | 0.9 | 0.01359 | no |
| BCCIP | 3 | 4.3 | 7 | 0.7 | 0.01428 | no |
| AR | 9 | 6.5 | 64 | 2.7 | 0.01595 | no |
| MSH6 | 7 | 5.1 | 46 | 1.9 | 0.0229 | no |
| CDK12 | 13 | 10 | 114 | 5.1 | 0.02444 | no |
| SPEN | 9 | 7.4 | 71 | 3.1 | 0.03814 | no |
| APC | 14 | 10.3 | 139 | 5.8 | 0.04331 | no |
| RAD51C | 3 | 2.3 | 12 | 0.5 | 0.04437 | no |
| BRCA1 | 6 | 4.3 | 43 | 1.8 | 0.04736 | no |
| BRE | 13 | 18.6 | 124 | 13 | 0.04899 | no |
| KMT2C | 24 | 19.8 | 278 | 12.4 | 0.05689 | no |
| CHD1 | 6 | 8.6 | 46 | 4.8 | 0.06043 | no |
| FAT3 | 22 | 30.1 | 262 | 27 | 0.07202 | no |
| TOPBP1 | 4 | 5.7 | 27 | 2.8 | 0.08459 | no |
| BRAF | 10 | 7.2 | 100 | 4.2 | 0.0869 | no |
| RAD51B | 19 | 14.6 | 226 | 9.4 | 0.10216 | no |
| RNF43 | 4 | 3.1 | 30 | 1.3 | 0.11024 | no |
| PMS2 | 3 | 2.2 | 19 | 0.8 | 0.11419 | no |
| PALB2 | 3 | 2.2 | 20 | 0.8 | 0.12634 | no |
| SPTA1 | 10 | 13.7 | 103 | 10.6 | 0.13149 | no |
| NCOR1 | 10 | 8.3 | 103 | 4.6 | 0.13149 | no |
| TMPRSS2 | 8 | 6 | 76 | 3.4 | 0.13367 | no |
| CHEK2 | 4 | 2.9 | 33 | 1.4 | 0.13892 | no |
| KDM6A | 11 | 8.1 | 119 | 5 | 0.15964 | no |
| HOXB13 | 2 | 1.7 | 12 | 0.6 | 0.17485 | no |
| PTEN | 14 | 10.1 | 170 | 7.1 | 0.17695 | no |
| ETV4 | 2 | 2.2 | 13 | 1.2 | 0.19492 | no |
| FOXA1 | 21 | 16.8 | 284 | 12.7 | 0.22727 | no |
| RAD50 | 3 | 2.3 | 29 | 1.3 | 0.25133 | no |
| ZFHX3 | 12 | 10.9 | 154 | 7.2 | 0.28727 | no |
| ATM | 9 | 6.5 | 107 | 4.5 | 0.28937 | no |
| RAD51 | 2 | 1.5 | 19 | 0.8 | 0.31829 | no |
| TP53 | 18 | 13 | 391 | 16.3 | 0.34294 | no |
| CDKN1B | 0 | 0 | 31 | 1.3 | 0.41096 | no |
| RAD51D | 1 | 0.8 | 9 | 0.4 | 0.42924 | no |
| MRE11A | 3 | 2.3 | 33 | 1.5 | 0.44325 | no |
| ATR | 6 | 4.6 | 75 | 3.3 | 0.44856 | no |
| MED12 | 4 | 3.1 | 50 | 2.2 | 0.53532 | no |
| ERF | 1 | 1 | 35 | 1.9 | 0.71961 | no |
| MSH2 | 3 | 2.2 | 43 | 1.8 | 0.73704 | no |
| RB1 | 3 | 2.2 | 47 | 2 | 0.75145 | no |
| SPOP | 16 | 12.3 | 275 | 12.2 | 0.89155 | no |
| EPCAM | 1 | 0.8 | 22 | 1 | 1 | no |
| PIK3CA | 5 | 3.6 | 99 | 4.1 | 1 | no |
| ZMYM3 | 1 | 1.4 | 31 | 3.2 | 1 | no |
| IDH1 | 2 | 1.4 | 35 | 1.5 | 1 | no |
| GEN1 | 1 | 1.2 | 21 | 2.2 | 1 | no |
| CTNNB1 | 3 | 2.2 | 68 | 2.8 | 1 | no |
| RAD51AP1 | 0 | 0 | 2 | 0.2 | 1 | no |
| ZNF350 | 0 | 0 | 2 | 0.2 | 1 | no |
| ABRA1 | 0 | 0 | 0 | 0 | N/A | no |
| BRCC36 | 0 | 0 | 0 | 0 | N/A | no |
| CTIP | 0 | 0 | 0 | 0 | N/A | no |
| DNAPK | 0 | 0 | 0 | 0 | N/A | no |
| EMSY | 0 | 0 | 0 | 0 | N/A | no |
| MERIT40 | 0 | 0 | 0 | 0 | N/A | no |
| MRE11 | 0 | 0 | 0 | 0 | N/A | no |
| NBS1 | 0 | 0 | 0 | 0 | N/A | no |
| POU5F1P1 | 0 | 0 | 0 | 0 | N/A | no |
| RAP80 | 0 | 0 | 0 | 0 | N/A | no |
| RASSF1A | 0 | 0 | 0 | 0 | N/A | no |

BRCA2^d^ = BRCA2-deficient; BRCA2^i^ = BRCA2-intact

**Supplementary Table 6**. Pathogenic SNV pathway enrichment in primary BRCA2^d^ and BRCA2^i^ tumors.

|  | BRCA2^d^ | | | BRCA2^i^ | | |  |
| --- | --- | --- | --- | --- | --- | --- | --- |
| Pathway | SNV Present  in Pathway (n) | SNV Absent  in Pathway (n) | Proportion of Samples Altered | SNV Present  in Pathway (n) | SNV Absent  in Pathway (n) | Proportion of Samples Altered | P-value |
| RTK-RAS | 11 | 59 | 16% | 120 | 834 | 13% | 0.46 |
| PI3K | 9 | 61 | 13% | 75 | 879 | 8% | 0.17 |
| WNT | 6 | 64 | 9% | 98 | 856 | 10% | 0.84 |
| Hippo | 8 | 62 | 11% | 118 | 836 | 12% | 1 |
| NOTCH | 4 | 66 | 6% | 94 | 860 | 10% | 0.4 |
| MYC | 1 | 69 | 1% | 18 | 936 | 2% | 1 |
| Cell Cycle | 0 | 70 | 0% | 18 | 936 | 2% | 0.63 |
| TGF-Beta | 0 | 70 | 0% | 16 | 938 | 2% | 0.63 |
| TP53 | 3 | 67 | 4% | 95 | 859 | 10% | 0.14 |
| NRF2 | 0 | 70 | 0% | 9 | 945 | 1% | 1 |
| Chromatin Remodeling* | 0 | 70 | 0% | 24 | 930 | 3% | 0.4 |

BRCA2^d^ = BRCA2-deficient; BRCA2^i^ = BRCA2-intact

*Pathway consists of SWI1, SWI2, SNF2, SWI3, SWI5, SWI6, HDAC1, HDAC2, RbAp46, RbAp48, MTA1, MTA2 / MTA3, MBD3, MBD2, CHD3, CHD4, INO80, SWR1

**Supplementary Table 7.** Pathogenic SNV frequency by BRCA2 status in metastatic tumors

| **Gene** | **SNVs**  **BRCA2^d^ (n)** | **SNV Freq.**  **BRCA2^d^ (%)** | **SNVs**  **BRCA2^i^ (n)** | **SNV Freq.**  **BRCA2^i^ (%)** | **Nominal**  **P-Value** | **Significant** |
| --- | --- | --- | --- | --- | --- | --- |
| KMT2D | 13 | 15.9 | 55 | 6.8 | 0.00876 | no |
| RAD51B | 2 | 2.7 | 0 | 0 | 0.00884 | no |
| APC | 13 | 15.3 | 59 | 7.2 | 0.0182 | no |
| RAD50 | 2 | 2.5 | 1 | 0.1 | 0.0249 | no |
| BRCA1 | 3 | 3.5 | 5 | 0.6 | 0.03221 | no |
| SPEN | 7 | 9.6 | 28 | 3.5 | 0.03954 | no |
| TP53 | 22 | 25.9 | 290 | 35.6 | 0.07384 | no |
| KMT2C | 10 | 13.7 | 53 | 6.6 | 0.07592 | no |
| RNF43 | 4 | 4.9 | 15 | 1.9 | 0.09608 | no |
| BRIP1 | 2 | 2.4 | 4 | 0.5 | 0.10314 | no |
| FOXA1 | 14 | 18.4 | 86 | 10.8 | 0.10379 | no |
| ETV1 | 2 | 2.5 | 5 | 0.6 | 0.13576 | no |
| MLH1 | 2 | 2.4 | 5 | 0.6 | 0.13576 | no |
| NBN | 2 | 2.4 | 6 | 0.7 | 0.17027 | no |
| ERG | 2 | 2.4 | 6 | 0.7 | 0.17027 | no |
| EPCAM | 1 | 1.3 | 1 | 0.1 | 0.18025 | no |
| HOXB13 | 1 | 1.3 | 1 | 0.1 | 0.18025 | no |
| ZFHX3 | 8 | 12.5 | 47 | 6.3 | 0.22829 | no |
| MSH2 | 2 | 2.4 | 8 | 1 | 0.24254 | no |
| NCOR1 | 0 | 0 | 22 | 2.7 | 0.25572 | no |
| ATR | 2 | 2.5 | 9 | 1.1 | 0.27929 | no |
| GRIN2A | 4 | 5.6 | 25 | 3.1 | 0.34417 | no |
| ASXL1 | 4 | 4.9 | 25 | 3.1 | 0.34417 | no |
| KDM6A | 4 | 4.9 | 25 | 3.1 | 0.34417 | no |
| CTNNB1 | 7 | 8.2 | 48 | 5.9 | 0.34708 | no |
| BRAF | 0 | 0 | 17 | 2.1 | 0.39371 | no |
| SPOP | 6 | 7.4 | 82 | 10.1 | 0.44736 | no |
| PMS2 | 1 | 1.2 | 5 | 0.6 | 0.44992 | no |
| PTEN | 6 | 7.1 | 80 | 9.8 | 0.56004 | no |
| RB1 | 2 | 2.4 | 38 | 4.7 | 0.57588 | no |
| TMPRSS2 | 1 | 1.2 | 8 | 1 | 0.59266 | no |
| CHEK2 | 1 | 1.2 | 8 | 1 | 0.59266 | no |
| ERF | 2 | 4.4 | 12 | 2.4 | 0.63408 | no |
| MGA | 3 | 4.2 | 22 | 3 | 0.72381 | no |
| AR | 8 | 9.8 | 70 | 8.6 | 0.83885 | no |
| CDK12 | 4 | 4.9 | 46 | 5.7 | 1 | no |
| RAD51D | 0 | 0 | 2 | 0.2 | 1 | no |
| RAD51C | 0 | 0 | 4 | 0.5 | 1 | no |
| ATM | 5 | 5.9 | 50 | 6.1 | 1 | no |
| CDKN1B | 1 | 1.2 | 13 | 1.6 | 1 | no |
| PALB2 | 1 | 1.2 | 10 | 1.2 | 1 | no |
| FANCA | 0 | 0 | 7 | 0.9 | 1 | no |
| MYC | 0 | 0 | 7 | 0.9 | 1 | no |
| MRE11A | 0 | 0 | 5 | 0.6 | 1 | no |
| MED12 | 2 | 2.5 | 19 | 2.3 | 1 | no |
| BARD1 | 0 | 0 | 6 | 0.7 | 1 | no |
| JAK1 | 3 | 3.7 | 32 | 4 | 1 | no |
| IDH1 | 0 | 0 | 3 | 0.4 | 1 | no |
| PIK3CA | 5 | 5.9 | 47 | 5.8 | 1 | no |
| MSH6 | 1 | 1.2 | 9 | 1.1 | 1 | no |
| RAD51 | 0 | 0 | 0 | 0 | N/A | no |

BRCA2^d^ = BRCA2-deficient; BRCA2^i^ = BRCA2-intact

**Supplementary Table 8.** Total SNV frequency by BRCA2 status in metastatic tumors

| **Gene** | **SNVs**  **BRCA2^d^ (n)** | **SNV Freq.**  **BRCA2^d^ (%)** | **SNVs**  **BRCA2^i^ (n)** | **SNV Freq.**  **BRCA2^i^ (%)** | **Nominal**  **P-Value** | **Significant** |
| --- | --- | --- | --- | --- | --- | --- |
| RAD51B | 6 | 8 | 6 | 0.7 | 0.00035 | yes |
| RAD50 | 3 | 3.8 | 1 | 0.1 | 0.00305 | yes |
| BRCA1 | 5 | 5.9 | 7 | 0.9 | 0.00313 | yes |
| KMT2D | 14 | 17.1 | 56 | 6.9 | 0.00444 | yes |
| BRIP1 | 5 | 6.1 | 8 | 1 | 0.00471 | yes |
| APC | 14 | 16.5 | 61 | 7.5 | 0.01121 | no |
| GRIN2A | 8 | 11.1 | 31 | 3.8 | 0.02445 | no |
| KMT2C | 12 | 16.4 | 56 | 6.9 | 0.02787 | no |
| ETV1 | 3 | 3.7 | 5 | 0.6 | 0.03221 | no |
| MSH2 | 4 | 4.8 | 10 | 1.2 | 0.03567 | no |
| NBN | 3 | 3.7 | 6 | 0.7 | 0.04508 | no |
| ATR | 4 | 4.9 | 11 | 1.4 | 0.04522 | no |
| SPEN | 7 | 9.6 | 29 | 3.6 | 0.07137 | no |
| TMPRSS2 | 3 | 3.7 | 8 | 1 | 0.07712 | no |
| ERG | 3 | 3.7 | 9 | 1.1 | 0.09602 | no |
| RNF43 | 4 | 4.9 | 15 | 1.9 | 0.09608 | no |
| ZFHX3 | 9 | 14.1 | 49 | 6.6 | 0.10594 | no |
| FOXA1 | 14 | 18.4 | 89 | 11.2 | 0.14982 | no |
| NCOR1 | 0 | 0 | 25 | 3.1 | 0.15898 | no |
| MLH1 | 2 | 2.4 | 6 | 0.7 | 0.17027 | no |
| HOXB13 | 1 | 1.3 | 1 | 0.1 | 0.18025 | no |
| MGA | 5 | 6.9 | 24 | 3.2 | 0.1837 | no |
| TP53 | 24 | 28.2 | 293 | 36 | 0.18912 | no |
| KDM6A | 5 | 6.1 | 27 | 3.3 | 0.2164 | no |
| CHEK2 | 2 | 2.4 | 8 | 1 | 0.24254 | no |
| EPCAM | 1 | 1.3 | 2 | 0.3 | 0.25793 | no |
| MSH6 | 2 | 2.4 | 9 | 1.1 | 0.27929 | no |
| CTNNB1 | 7 | 8.2 | 48 | 5.9 | 0.34708 | no |
| JAK1 | 5 | 6.2 | 32 | 4 | 0.38478 | no |
| PMS2 | 1 | 1.2 | 5 | 0.6 | 0.44992 | no |
| ATM | 7 | 8.2 | 51 | 6.3 | 0.48418 | no |
| ASXL1 | 4 | 4.9 | 26 | 3.2 | 0.51807 | no |
| BARD1 | 1 | 1.2 | 8 | 1 | 0.59266 | no |
| CDK12 | 6 | 7.4 | 47 | 5.8 | 0.62663 | no |
| ERF | 2 | 4.4 | 12 | 2.4 | 0.63408 | no |
| RB1 | 3 | 3.5 | 39 | 4.8 | 0.78968 | no |
| AR | 8 | 9.8 | 71 | 8.7 | 0.83998 | no |
| SPOP | 8 | 9.9 | 84 | 10.4 | 1 | no |
| CDKN1B | 1 | 1.2 | 13 | 1.6 | 1 | no |
| IDH1 | 0 | 0 | 4 | 0.5 | 1 | no |
| RAD51D | 0 | 0 | 2 | 0.2 | 1 | no |
| BRAF | 1 | 1.2 | 17 | 2.1 | 1 | no |
| MED12 | 2 | 2.5 | 19 | 2.3 | 1 | no |
| PIK3CA | 5 | 5.9 | 49 | 6 | 1 | no |
| PALB2 | 1 | 1.2 | 10 | 1.2 | 1 | no |
| FANCA | 0 | 0 | 7 | 0.9 | 1 | no |
| MYC | 0 | 0 | 7 | 0.9 | 1 | no |
| MRE11A | 0 | 0 | 6 | 0.7 | 1 | no |
| RAD51C | 0 | 0 | 4 | 0.5 | 1 | no |
| PTEN | 8 | 9.4 | 81 | 10 | 1 | no |
| RAD51 | 0 | 0 | 0 | 0 | N/A | no |

BRCA2^d^ = BRCA2-deficient; BRCA2^i^ = BRCA2-intact

**Supplementary Table 9.** Pathogenic SNV frequency in primary versus metastatic *BRCA2^d^* tumors

| **Gene** | **SNVs**  **Primary (n)** | **SNV Freq.**  **Primary (%)** | **SNVs**  **Metastatic (n)** | **SNV Freq.**  **Metastatic (%)** | **Nominal**  **P-Value** | **Significant** |
| --- | --- | --- | --- | --- | --- | --- |
| FOXA1 | 3 | 5.45 | 14 | 19.44 | 0.033226 | no |
| AR | 1 | 1.47 | 8 | 10.26 | 0.037196 | no |
| NCOR1 | 3 | 6 | 0 | 0 | 0.075347 | no |
| BRAF | 3 | 4.41 | 0 | 0 | 0.09276 | no |
| MYC | 3 | 4.41 | 0 | 0 | 0.098638 | no |
| APC | 5 | 7.58 | 13 | 16.05 | 0.136426 | no |
| BARD1 | 2 | 3.45 | 0 | 0 | 0.182753 | no |
| IDH1 | 2 | 2.94 | 0 | 0 | 0.206603 | no |
| FANCA | 2 | 2.94 | 0 | 0 | 0.21521 | no |
| ZFHX3 | 2 | 5 | 8 | 13.33 | 0.307651 | no |
| CDK12 | 6 | 10 | 4 | 5.19 | 0.333064 | no |
| MSH6 | 3 | 4.41 | 1 | 1.28 | 0.338577 | no |
| RAD51 | 1 | 1.67 | 0 | 0 | 0.437956 | no |
| MRE11A | 1 | 1.67 | 0 | 0 | 0.437956 | no |
| CTNNB1 | 2 | 2.94 | 5 | 6.17 | 0.454808 | no |
| PIK3CA | 2 | 2.94 | 5 | 6.17 | 0.454808 | no |
| RAD51B | 0 | 0 | 2 | 2.82 | 0.499706 | no |
| MSH2 | 0 | 0 | 2 | 2.5 | 0.499908 | no |
| TP53 | 14 | 20.59 | 21 | 25.93 | 0.561065 | no |
| PTEN | 7 | 10.29 | 6 | 7.41 | 0.571763 | no |
| KMT2C | 5 | 9.8 | 10 | 14.49 | 0.579606 | no |
| PALB2 | 2 | 2.94 | 1 | 1.25 | 0.594103 | no |
| PMS2 | 2 | 2.94 | 1 | 1.28 | 0.598087 | no |
| BRCA1 | 1 | 1.47 | 3 | 3.7 | 0.625724 | no |
| MED12 | 3 | 5 | 2 | 2.6 | 0.653408 | no |
| RNF43 | 4 | 6.67 | 4 | 5.19 | 0.729479 | no |
| SPOP | 3 | 5 | 6 | 7.79 | 0.731052 | no |
| KMT2D | 12 | 18.18 | 13 | 16.67 | 0.828697 | no |
| ATM | 4 | 5.88 | 5 | 6.17 | 1 | no |
| ATR | 2 | 3.33 | 2 | 2.6 | 1 | no |
| BRIP1 | 1 | 1.52 | 2 | 2.56 | 1 | no |
| CDKN1B | 0 | 0 | 1 | 1.28 | 1 | no |
| CHEK2 | 1 | 1.47 | 1 | 1.28 | 1 | no |
| EPCAM | 1 | 1.82 | 1 | 1.39 | 1 | no |
| ERG | 1 | 1.59 | 1 | 1.28 | 1 | no |
| ETV1 | 1 | 1.52 | 2 | 2.6 | 1 | no |
| HOXB13 | 1 | 1.96 | 1 | 1.37 | 1 | no |
| MLH1 | 2 | 2.94 | 2 | 2.47 | 1 | no |
| NBN | 1 | 1.47 | 2 | 2.56 | 1 | no |
| RAD50 | 1 | 1.67 | 2 | 2.63 | 1 | no |
| RAD51C | 0 | 0 | 0 | 0 | 1 | no |
| RAD51D | 0 | 0 | 0 | 0 | 1 | no |
| RB1 | 1 | 1.47 | 1 | 1.23 | 1 | no |
| TMPRSS2 | 1 | 1.59 | 1 | 1.28 | 1 | no |

**Supplementary Table 10.** COSMIC signatures of known etiology with cosine similarity >0.9 to detected signatures in primary *BRCA2*-deficient (*BRCA2^d^*) and *BRCA2*-intact (*BRCA2^i^*) tumors from ICGC and TCGA.

| **Signature Type** | ***BRCA2^d^* Tumors** | ***BRCA2^i^* Tumors** |
| --- | --- | --- |
| **SBS** | Found Sig3 most similar to SBS1  Etiology: Spontaneous deamination of 5-methyl-cystosine (clock-like signature) [similarity: 0.931] | Found Sig3 most similar to SBS1  Etiology: Spontaneous deamination of 5-methyl-cystosine (clock-like signature) [similarity: 0.993  Found Sig6 most similar to SBS15  Etiology: Defective DNA mismatch repair [similarity: 0.914] |
| **DBS** | -- | -- |
| **ID** | Found Sig1 most similar to ID1  Etiology: Slippage during DNA replication of the replicated DNA strand [similarity: 0.966]  Found Sig2 most similar to ID1  Etiology: Slippage during DNA replication of the replicated DNA strand [similarity: 0.935]  Found Sig4 most similar to ID6  Etiology: Defective homologous recombination DNA damage repair [similarity: 0.962] | Found Sig1 most similar to ID1  Etiology: Slippage during DNA replication of the replicated DNA strand [similarity: 0.999]  Found Sig4 most similar to ID2  Etiology: Slippage during DNA replication of the replicated DNA strand [similarity: 0.998] |

SBS = Single Base Substitution, DBS = Doublet Base Substitutions, ID = Small Insertions and Deletions

**Supplementary Table 11**. CNA frequency by BRCA2 status in primary tumors

Homozygous Deletion

| **Gene** | **BRCA2^d^ (n)** | **Frequency**  **BRCA2^d^ (%)** | **BRCA2^i^ (n)** | **Frequency**  **BRCA2^i^ (%)** | **Nominal P-Value** | **Significant** |
| --- | --- | --- | --- | --- | --- | --- |
| AK9 | 7 | 10 | 34 | 3.56 | 0.017675119 | no |
| APC | 5 | 3.67 | 9 | 0.38 | 0.000559466 | yes |
| AR | 0 | 0 | 0 | 0 | 1 | no |
| ATM | 2 | 1.45 | 10 | 0.42 | 0.13583235 | no |
| ATR | 0 | 0 | 2 | 0.09 | 1 | no |
| BACH1 | 0 | 0 | 2 | 0.21 | 1 | no |
| BARD1 | 0 | 0 | 2 | 0.09 | 1 | no |
| BCCIP | 0 | 0 | 4 | 0.42 | 1 | no |
| BRAF | 0 | 0 | 0 | 0 | 1 | no |
| BRCA1 | 0 | 0 | 6 | 0.25 | 1 | no |
| BRE | 0 | 0 | 1 | 0.1 | 1 | no |
| BRIP1 | 0 | 0 | 0 | 0 | 1 | no |
| CDK12 | 0 | 0 | 3 | 0.13 | 1 | no |
| CDKN1B | 6 | 4.35 | 35 | 1.46 | 0.021657649 | no |
| CHD1 | 4 | 5.72 | 30 | 3.14 | 0.284005132 | no |
| CHEK2 | 0 | 0 | 8 | 0.33 | 1 | no |
| CNBD1 | 0 | 0 | 2 | 0.21 | 1 | no |
| CPQ | 0 | 0 | 0 | 0 | 1 | no |
| CSMD3 | 1 | 1.43 | 8 | 0.84 | 0.472295174 | no |
| CTNNB1 | 0 | 0 | 1 | 0.04 | 1 | no |
| DCAF13 | 0 | 0 | 0 | 0 | 1 | no |
| EPCAM | 1 | 0.8 | 5 | 0.22 | 0.278623176 | no |
| ERF | 1 | 1.02 | 13 | 0.72 | 0.522504344 | no |
| ERG | 0 | 0 | 49 | 2.17 | 0.110442567 | no |
| ETV1 | 0 | 0 | 4 | 0.17 | 1 | no |
| ETV4 | 0 | 0 | 11 | 0.98 | 1 | no |
| FANCA | 3 | 2.17 | 27 | 1.13 | 0.221601151 | no |
| FOXA1 | 0 | 0 | 1 | 0.04 | 1 | no |
| FOXO3 | 6 | 8.57 | 34 | 3.56 | 0.049136657 | no |
| GEN1 | 0 | 0 | 1 | 0.1 | 1 | no |
| HOXB13 | 0 | 0 | 0 | 0 | 1 | no |
| IDH1 | 0 | 0 | 2 | 0.08 | 1 | no |
| KCNB2 | 1 | 1.43 | 2 | 0.21 | 0.191378674 | no |
| KLLN | 2 | 2.86 | 58 | 6.07 | 0.425419331 | no |
| KMT2C | 0 | 0 | 5 | 0.22 | 1 | no |
| KMT2D | 0 | NA | 0 | 0 | 1 | no |
| LINC00535 | 0 | 0 | 0 | 0 | 1 | no |
| MAP3K7 | 7 | 10 | 38 | 3.98 | 0.02862061 | no |
| MED12 | 0 | 0 | 1 | 0.04 | 1 | no |
| MLH1 | 0 | 0 | 1 | 0.04 | 1 | no |
| MRE11A | 0 | 0 | 2 | 0.09 | 1 | no |
| MSH2 | 1 | 0.72 | 7 | 0.29 | 0.361261741 | no |
| MSH6 | 0 | 0 | 5 | 0.21 | 1 | no |
| MYC | 0 | 0 | 2 | 0.08 | 1 | no |
| NBN | 0 | 0 | 3 | 0.13 | 1 | no |
| NCOR1 | 0 | 0 | 3 | 0.13 | 1 | no |
| NCOR2 | 1 | 1.43 | 6 | 0.63 | 0.391442694 | no |
| NEK3 | 14 | 20 | 32 | 3.35 | 5.62E-07 | yes |
| NSMCE2 | 0 | 0 | 0 | 0 | 1 | no |
| PALB2 | 0 | 0 | 1 | 0.04 | 1 | no |
| PIK3CA | 0 | 0 | 5 | 0.21 | 1 | no |
| PMS2 | 0 | 0 | 2 | 0.08 | 1 | no |
| PTEN | 5 | 3.62 | 193 | 8.05 | 0.070448796 | no |
| RAD50 | 2 | 1.54 | 3 | 0.13 | 0.0264927 | no |
| RAD51 | 0 | 0 | 5 | 0.22 | 1 | no |
| RAD51AP1 | 1 | 1.43 | 0 | 0 | 0.068292683 | no |
| RAD51B | 0 | 0 | 7 | 0.29 | 1 | no |
| RAD51C | 0 | 0 | 3 | 0.13 | 1 | no |
| RAD51D | 0 | 0 | 3 | 0.13 | 1 | no |
| RAD54B | 0 | 0 | 1 | 0.1 | 1 | no |
| RB1 | 16 | 11.59 | 50 | 2.09 | 2.06E-07 | yes |
| RNF43 | 0 | 0 | 6 | 0.27 | 1 | no |
| RSPO2 | 2 | 2.5 | 0 | 0 | 0.005716084 | no |
| RUNX1T1 | 0 | 0 | 0 | 0 | 1 | no |
| SNX31 | 0 | 0 | 0 | 0 | 1 | no |
| SPOP | 0 | 0 | 1 | 0.04 | 1 | no |
| TMPRSS2 | 2 | 1.5 | 61 | 2.71 | 0.579629545 | no |
| TOPBP1 | 0 | 0 | 1 | 0.1 | 1 | no |
| TP53 | 2 | 1.45 | 30 | 1.25 | 0.692178937 | no |
| TPD52 | 0 | 0 | 2 | 0.21 | 1 | no |
| UBR5 | 0 | 0 | 0 | 0 | 1 | no |
| WISP3 | 6 | 8.11 | 33 | 3.4 | 0.051415976 | no |
| ZFHX3 | 2 | 1.82 | 39 | 1.82 | 1 | no |
| ZMYM3 | 0 | 0 | 1 | 0.1 | 1 | no |
| ZNF350 | 0 | 0 | 0 | 0 | 1 | no |

LOH

| **Gene** | **BRCA2^d^ (n)** | **Frequency**  **BRCA2^d^ (%)** | **BRCA2^i^ (n)** | **Frequency**  **BRCA2^i^ (%)** | **Nominal P-Value** | **Significant** |
| --- | --- | --- | --- | --- | --- | --- |
| AK9 | 4 | 5.71 | 111 | 11.63 | 0.168329377 | no |
| APC | 1 | 0.74 | 57 | 2.38 | 0.369369562 | no |
| AR | 1 | 0.72 | 29 | 1.21 | 1 | no |
| ATM | 4 | 2.9 | 43 | 1.79 | 0.321308846 | no |
| ATR | 0 | 0 | 8 | 0.35 | 1 | no |
| BACH1 | 1 | 1.37 | 29 | 2.99 | 0.716686192 | no |
| BARD1 | 1 | 0.78 | 23 | 1.02 | 1 | no |
| BCCIP | 1 | 1.43 | 65 | 6.8 | 0.079561652 | no |
| BRAF | 1 | 0.72 | 8 | 0.33 | 0.396129573 | no |
| BRCA1 | 1 | 0.72 | 57 | 2.37 | 0.371204499 | no |
| BRE | 1 | 1.43 | 21 | 2.2 | 1 | no |
| BRIP1 | 1 | 0.74 | 19 | 0.79 | 1 | no |
| CDK12 | 2 | 1.54 | 36 | 1.59 | 1 | no |
| CDKN1B | 1 | 0.72 | 110 | 4.58 | 0.029040692 | no |
| CHD1 | 2 | 2.86 | 72 | 7.54 | 0.226239139 | no |
| CHEK2 | 2 | 1.45 | 59 | 2.46 | 0.771655845 | no |
| CNBD1 | 0 | 0 | 10 | 1.05 | 1 | no |
| CPQ | 0 | 0 | 5 | 0.52 | 1 | no |
| CSMD3 | 0 | 0 | 10 | 1.05 | 1 | no |
| CTNNB1 | 0 | 0 | 9 | 0.38 | 1 | no |
| DCAF13 | 0 | 0 | 2 | 0.21 | 1 | no |
| EPCAM | 2 | 1.6 | 29 | 1.3 | 0.678176333 | no |
| ERF | 2 | 2.04 | 38 | 2.09 | 1 | no |
| ERG | 4 | 3.01 | 79 | 3.5 | 1 | no |
| ETV1 | 1 | 0.74 | 9 | 0.38 | 0.424835754 | no |
| ETV4 | 1 | 1.12 | 64 | 5.73 | 0.08249521 | no |
| FANCA | 13 | 9.42 | 172 | 7.17 | 0.312095177 | no |
| FOXA1 | 1 | 0.8 | 28 | 1.25 | 1 | no |
| FOXO3 | 4 | 5.72 | 111 | 11.62 | 0.168329377 | no |
| GEN1 | 1 | 1.25 | 22 | 2.27 | 1 | no |
| HOXB13 | 1 | 0.83 | 18 | 0.83 | 1 | no |
| IDH1 | 1 | 0.72 | 21 | 0.88 | 1 | no |
| KCNB2 | 0 | 0 | 4 | 0.42 | 1 | no |
| KLLN | 6 | 8.57 | 88 | 9.21 | 1 | no |
| KMT2C | 1 | 0.83 | 21 | 0.93 | 1 | no |
| KMT2D | 0 | NA | 23 | 0.96 | 0.631934478 | no |
| LINC00535 | 0 | 0 | 8 | 0.84 | 1 | no |
| MAP3K7 | 5 | 7.14 | 126 | 13.2 | 0.19228903 | no |
| MED12 | 1 | 0.77 | 28 | 1.24 | 1 | no |
| MLH1 | 0 | 0 | 11 | 0.46 | 1 | no |
| MRE11A | 1 | 0.77 | 13 | 0.58 | 0.544744431 | no |
| MSH2 | 2 | 1.45 | 30 | 1.25 | 0.692178937 | no |
| MSH6 | 1 | 0.72 | 30 | 1.25 | 1 | no |
| MYC | 0 | 0 | 3 | 0.13 | 1 | no |
| NBN | 0 | 0 | 7 | 0.29 | 1 | no |
| NCOR1 | 2 | 1.67 | 81 | 3.59 | 0.438374189 | no |
| NCOR2 | 1 | 1.43 | 36 | 3.77 | 0.507633965 | no |
| NEK3 | 7 | 10 | 156 | 16.33 | 0.179167995 | no |
| NSMCE2 | 0 | 0 | 5 | 0.52 | 1 | no |
| PALB2 | 1 | 0.72 | 31 | 1.29 | 1 | no |
| PIK3CA | 0 | 0 | 6 | 0.25 | 1 | no |
| PMS2 | 0 | 0 | 5 | 0.21 | 1 | no |
| PTEN | 6 | 4.35 | 88 | 3.67 | 0.641001484 | no |
| RAD50 | 0 | 0 | 34 | 1.51 | 0.257250473 | no |
| RAD51 | 2 | 1.54 | 59 | 2.61 | 0.771637788 | no |
| RAD51AP1 | 1 | 1.43 | 67 | 7.02 | 0.079499231 | no |
| RAD51B | 2 | 1.54 | 42 | 1.75 | 1 | no |
| RAD51C | 2 | 1.54 | 26 | 1.15 | 0.663011582 | no |
| RAD51D | 2 | 1.54 | 37 | 1.64 | 1 | no |
| RAD54B | 0 | 0 | 8 | 0.84 | 1 | no |
| RB1 | 10 | 7.25 | 187 | 7.8 | 1 | no |
| RNF43 | 2 | 1.54 | 30 | 1.33 | 0.692556538 | no |
| RSPO2 | 0 | 0 | 7 | 0.72 | 1 | no |
| RUNX1T1 | 0 | 0 | 8 | 0.82 | 1 | no |
| SNX31 | 0 | 0 | 1 | 0.1 | 1 | no |
| SPOP | 1 | 0.77 | 20 | 0.89 | 1 | no |
| TMPRSS2 | 4 | 3.01 | 100 | 4.44 | 0.659167488 | no |
| TOPBP1 | 0 | 0 | 10 | 1.05 | 1 | no |
| TP53 | 8 | 5.8 | 152 | 6.34 | 1 | no |
| TPD52 | 0 | 0 | 4 | 0.42 | 1 | no |
| UBR5 | 0 | 0 | 2 | 0.21 | 1 | no |
| WISP3 | 4 | 5.4 | 107 | 11.02 | 0.169241829 | no |
| ZFHX3 | 8 | 7.27 | 158 | 7.38 | 1 | no |
| ZMYM3 | 1 | 1.43 | 28 | 2.93 | 0.715492013 | no |
| ZNF350 | 2 | 2.86 | 18 | 1.88 | 0.641521765 | no |

Gain

| **Gene** | **BRCA2^d^ (n)** | **Frequency**  **BRCA2^d^ (%)** | **BRCA2^i^ (n)** | **Frequency**  **BRCA2^i^ (%)** | **Nominal P-Value** | **Significant** |
| --- | --- | --- | --- | --- | --- | --- |
| AK9 | 0 | 0 | 4 | 0.42 | 1 | no |
| APC | 2 | 1.47 | 14 | 0.58 | 0.210675972 | no |
| AR | 0 | 0 | 13 | 0.54 | 1 | no |
| ATM | 1 | 0.72 | 32 | 1.33 | 1 | no |
| ATR | 7 | 5.38 | 62 | 2.75 | 0.098267586 | no |
| BACH1 | 1 | 1.37 | 20 | 2.06 | 1 | no |
| BARD1 | 2 | 1.56 | 8 | 0.35 | 0.097118404 | no |
| BCCIP | 0 | 0 | 8 | 0.84 | 1 | no |
| BRAF | 8 | 5.8 | 95 | 3.96 | 0.267006856 | no |
| BRCA1 | 2 | 1.45 | 7 | 0.29 | 0.082319084 | no |
| BRE | 3 | 4.28 | 15 | 1.57 | 0.119024867 | no |
| BRIP1 | 5 | 3.68 | 16 | 0.67 | 0.004191867 | no |
| CDK12 | 2 | 1.54 | 8 | 0.35 | 0.099597031 | no |
| CDKN1B | 0 | 0 | 10 | 0.42 | 1 | no |
| CHD1 | 2 | 2.86 | 7 | 0.73 | 0.121234941 | no |
| CHEK2 | 0 | 0 | 3 | 0.13 | 1 | no |
| CNBD1 | 10 | 14.29 | 101 | 10.57 | 0.320423097 | no |
| CPQ | 10 | 14.28 | 107 | 11.21 | 0.435201056 | no |
| CSMD3 | 9 | 12.86 | 105 | 11 | 0.559826001 | no |
| CTNNB1 | 3 | 2.17 | 38 | 1.59 | 0.486663139 | no |
| DCAF13 | 10 | 14.28 | 109 | 11.42 | 0.441342548 | no |
| EPCAM | 1 | 0.8 | 13 | 0.58 | 0.533913375 | no |
| ERF | 1 | 1.02 | 6 | 0.33 | 0.30850751 | no |
| ERG | 4 | 3.01 | 17 | 0.75 | 0.026262602 | no |
| ETV1 | 12 | 8.82 | 100 | 4.17 | 0.016973862 | no |
| ETV4 | 2 | 2.25 | 6 | 0.54 | 0.112710309 | no |
| FANCA | 0 | 0 | 5 | 0.21 | 1 | no |
| FOXA1 | 3 | 2.4 | 12 | 0.54 | 0.04126903 | no |
| FOXO3 | 0 | 0 | 2 | 0.21 | 1 | no |
| GEN1 | 4 | 5 | 14 | 1.44 | 0.042017855 | no |
| HOXB13 | 7 | 5.78 | 13 | 0.6 | 4.36E-05 | yes |
| IDH1 | 3 | 2.17 | 9 | 0.38 | 0.024145118 | no |
| KCNB2 | 8 | 11.43 | 104 | 10.89 | 0.843170149 | no |
| KLLN | 0 | 0 | 6 | 0.63 | 1 | no |
| KMT2C | 5 | 4.13 | 84 | 3.73 | 0.80393051 | no |
| KMT2D | 0 | NA | 17 | 0.71 | 1 | no |
| LINC00535 | 11 | 15.71 | 103 | 10.79 | 0.23403278 | no |
| MAP3K7 | 0 | 0 | 3 | 0.31 | 1 | no |
| MED12 | 0 | 0 | 11 | 0.49 | 1 | no |
| MLH1 | 3 | 2.17 | 38 | 1.59 | 0.486663139 | no |
| MRE11A | 2 | 1.54 | 34 | 1.51 | 1 | no |
| MSH2 | 1 | 0.72 | 13 | 0.54 | 0.544069229 | no |
| MSH6 | 1 | 0.72 | 13 | 0.54 | 0.544069229 | no |
| MYC | 15 | 10.87 | 121 | 5.05 | 0.009605149 | no |
| NBN | 15 | 10.87 | 123 | 5.13 | 0.010097816 | no |
| NCOR1 | 1 | 0.83 | 10 | 0.44 | 0.435634795 | no |
| NCOR2 | 2 | 2.86 | 20 | 2.1 | 0.657986568 | no |
| NEK3 | 0 | 0 | 3 | 0.31 | 1 | no |
| NSMCE2 | 7 | 10 | 106 | 11.1 | 1 | no |
| PALB2 | 2 | 1.45 | 30 | 1.25 | 0.692178937 | no |
| PIK3CA | 6 | 4.35 | 64 | 2.67 | 0.275040792 | no |
| PMS2 | 13 | 9.42 | 97 | 4.04 | 0.007582202 | no |
| PTEN | 0 | 0 | 5 | 0.21 | 1 | no |
| RAD50 | 3 | 2.31 | 12 | 0.53 | 0.044577143 | no |
| RAD51 | 0 | 0 | 7 | 0.31 | 1 | no |
| RAD51AP1 | 1 | 1.43 | 12 | 1.26 | 0.60354806 | no |
| RAD51B | 0 | 0 | 12 | 0.5 | 1 | no |
| RAD51C | 3 | 2.31 | 11 | 0.49 | 0.037024812 | no |
| RAD51D | 1 | 0.77 | 11 | 0.49 | 0.490431224 | no |
| RAD54B | 13 | 18.57 | 103 | 10.79 | 0.074990078 | no |
| RB1 | 0 | 0 | 3 | 0.13 | 1 | no |
| RNF43 | 3 | 2.31 | 11 | 0.49 | 0.037024812 | no |
| RSPO2 | 11 | 13.75 | 107 | 11.01 | 0.460124858 | no |
| RUNX1T1 | 13 | 16.05 | 104 | 10.71 | 0.14251653 | no |
| SNX31 | 10 | 14.28 | 107 | 11.2 | 0.435201056 | no |
| SPOP | 4 | 3.08 | 12 | 0.53 | 0.009189715 | no |
| TMPRSS2 | 6 | 4.51 | 16 | 0.71 | 0.000949389 | yes |
| TOPBP1 | 8 | 11.43 | 60 | 6.28 | 0.127936194 | no |
| TP53 | 0 | 0 | 1 | 0.04 | 1 | no |
| TPD52 | 10 | 14.28 | 103 | 10.79 | 0.328582025 | no |
| UBR5 | 10 | 14.28 | 109 | 11.41 | 0.441342548 | no |
| WISP3 | 0 | 0 | 3 | 0.31 | 1 | no |
| ZFHX3 | 2 | 1.82 | 2 | 0.09 | 0.013287978 | no |
| ZMYM3 | 0 | 0 | 11 | 1.15 | 1 | no |
| ZNF350 | 2 | 2.86 | 14 | 1.46 | 0.299440136 | no |

Amplification

| **Gene** | **BRCA2^d^ (n)** | **Frequency**  **BRCA2^d^ (%)** | **BRCA2^i^ (n)** | **Frequency**  **BRCA2^i^ (%)** | **Nominal P-Value** | **Significant** |
| --- | --- | --- | --- | --- | --- | --- |
| AK9 | 0 | 0 | 1 | 0.1 | 1 | no |
| APC | 0 | 0 | 1 | 0.04 | 1 | no |
| AR | 4 | 2.9 | 16 | 0.67 | 0.020573419 | no |
| ATM | 0 | 0 | 3 | 0.13 | 1 | no |
| ATR | 1 | 0.77 | 6 | 0.27 | 0.324871758 | no |
| BACH1 | 0 | 0 | 1 | 0.1 | 1 | no |
| BARD1 | 0 | 0 | 0 | 0 | 1 | no |
| BCCIP | 1 | 1.43 | 2 | 0.21 | 0.191378674 | no |
| BRAF | 0 | 0 | 9 | 0.38 | 1 | no |
| BRCA1 | 0 | 0 | 3 | 0.12 | 1 | no |
| BRE | 0 | 0 | 2 | 0.21 | 1 | no |
| BRIP1 | 0 | 0 | 8 | 0.33 | 1 | no |
| CDK12 | 0 | 0 | 2 | 0.09 | 1 | no |
| CDKN1B | 0 | 0 | 6 | 0.25 | 1 | no |
| CHD1 | 0 | 0 | 1 | 0.1 | 1 | no |
| CHEK2 | 0 | 0 | 1 | 0.04 | 1 | no |
| CNBD1 | 0 | 0 | 38 | 3.98 | 0.103982125 | no |
| CPQ | 1 | 1.43 | 38 | 3.98 | 0.511969829 | no |
| CSMD3 | 2 | 2.86 | 38 | 3.98 | 1 | no |
| CTNNB1 | 0 | 0 | 2 | 0.08 | 1 | no |
| DCAF13 | 1 | 1.43 | 37 | 3.88 | 0.509401384 | no |
| EPCAM | 0 | 0 | 1 | 0.04 | 1 | no |
| ERF | 0 | 0 | 0 | 0 | 1 | no |
| ERG | 0 | 0 | 1 | 0.04 | 1 | no |
| ETV1 | 2 | 1.47 | 5 | 0.21 | 0.050345745 | no |
| ETV4 | 0 | 0 | 0 | 0 | 1 | no |
| FANCA | 0 | 0 | 1 | 0.04 | 1 | no |
| FOXA1 | 1 | 0.8 | 53 | 2.37 | 0.363898899 | no |
| FOXO3 | 0 | 0 | 1 | 0.1 | 1 | no |
| GEN1 | 0 | 0 | 1 | 0.1 | 1 | no |
| HOXB13 | 0 | 0 | 13 | 0.6 | 1 | no |
| IDH1 | 0 | 0 | 1 | 0.04 | 1 | no |
| KCNB2 | 0 | 0 | 37 | 3.87 | 0.170957978 | no |
| KLLN | 0 | 0 | 1 | 0.1 | 1 | no |
| KMT2C | 0 | 0 | 5 | 0.22 | 1 | no |
| KMT2D | 0 | NA | 1 | 0.04 | 1 | no |
| LINC00535 | 0 | 0 | 41 | 4.29 | 0.106323624 | no |
| MAP3K7 | 0 | 0 | 0 | 0 | 1 | no |
| MED12 | 1 | 0.77 | 0 | 0 | 0.054507338 | no |
| MLH1 | 0 | 0 | 0 | 0 | 1 | no |
| MRE11A | 0 | 0 | 2 | 0.09 | 1 | no |
| MSH2 | 0 | 0 | 1 | 0.04 | 1 | no |
| MSH6 | 0 | 0 | 1 | 0.04 | 1 | no |
| MYC | 8 | 5.8 | 71 | 2.96 | 0.073581497 | no |
| NBN | 5 | 3.62 | 56 | 2.33 | 0.380946095 | no |
| NCOR1 | 0 | 0 | 0 | 0 | 1 | no |
| NCOR2 | 0 | 0 | 3 | 0.31 | 1 | no |
| NEK3 | 0 | 0 | 1 | 0.1 | 1 | no |
| NSMCE2 | 3 | 4.29 | 35 | 3.67 | 0.740005342 | no |
| PALB2 | 1 | 0.72 | 4 | 0.17 | 0.244210288 | no |
| PIK3CA | 1 | 0.72 | 13 | 0.54 | 0.544069229 | no |
| PMS2 | 1 | 0.72 | 2 | 0.08 | 0.154584506 | no |
| PTEN | 0 | 0 | 2 | 0.08 | 1 | no |
| RAD50 | 0 | 0 | 1 | 0.04 | 1 | no |
| RAD51 | 0 | 0 | 1 | 0.04 | 1 | no |
| RAD51AP1 | 0 | 0 | 1 | 0.1 | 1 | no |
| RAD51B | 0 | 0 | 2 | 0.08 | 1 | no |
| RAD51C | 0 | 0 | 1 | 0.04 | 1 | no |
| RAD51D | 0 | 0 | 2 | 0.09 | 1 | no |
| RAD54B | 0 | 0 | 40 | 4.19 | 0.104892567 | no |
| RB1 | 0 | 0 | 0 | 0 | 1 | no |
| RNF43 | 0 | 0 | 1 | 0.04 | 1 | no |
| RSPO2 | 2 | 2.5 | 36 | 3.7 | 1 | no |
| RUNX1T1 | 0 | 0 | 39 | 4.02 | 0.066591045 | no |
| SNX31 | 1 | 1.43 | 41 | 4.29 | 0.355300598 | no |
| SPOP | 0 | 0 | 4 | 0.18 | 1 | no |
| TMPRSS2 | 0 | 0 | 18 | 0.8 | 0.620130306 | no |
| TOPBP1 | 1 | 1.43 | 2 | 0.21 | 0.191378674 | no |
| TP53 | 0 | 0 | 0 | 0 | 1 | no |
| TPD52 | 1 | 1.43 | 37 | 3.87 | 0.509401384 | no |
| UBR5 | 1 | 1.43 | 38 | 3.98 | 0.511969829 | no |
| WISP3 | 0 | 0 | 1 | 0.1 | 1 | no |
| ZFHX3 | 0 | 0 | 0 | 0 | 1 | no |
| ZMYM3 | 1 | 1.43 | 0 | 0 | 0.068292683 | no |
| ZNF350 | 0 | 0 | 3 | 0.31 | 1 | no |

**Supplementary Table 12**. CNA frequency by BRCA2 status in metastatic tumors

Homozygous Deletion

| **Gene** | **BRCA2^d^ (n)** | **Frequency**  **BRCA2^d^ (%)** | **BRCA2^i^ (n)** | **Frequency**  **BRCA2^i^ (%)** | **Nominal P-Value** | **Significant** |
| --- | --- | --- | --- | --- | --- | --- |
| AGO2 | 0 | 0 | 1 | 0.2 | 1 | no |
| AMER1 | 1 | 1.37 | 2 | 0.25 | 0.22831563 | no |
| APC | 4 | 4.7 | 26 | 3.19 | 0.518065434 | no |
| AR | 0 | 0 | 1 | 0.12 | 1 | no |
| ATM | 2 | 2.35 | 6 | 0.74 | 0.170274657 | no |
| ATR | 0 | 0 | 0 | 0 | 1 | no |
| BARD1 | 0 | 0 | 2 | 0.25 | 1 | no |
| BRAF | 0 | 0 | 1 | 0.12 | 1 | no |
| BRCA1 | 0 | 0 | 1 | 0.12 | 1 | no |
| BRIP1 | 0 | 0 | 0 | 0 | 1 | no |
| CDK12 | 0 | 0 | 5 | 0.62 | 1 | no |
| CDKN1B | 2 | 2.44 | 45 | 5.53 | 0.304686895 | no |
| CHEK2 | 0 | 0 | 5 | 0.61 | 1 | no |
| CTNNB1 | 0 | 0 | 0 | 0 | 1 | no |
| CYSLTR2 | 7 | 15.56 | 19 | 3.76 | 0.003073999 | no |
| DUSP4 | 3 | 6.67 | 26 | 5.15 | 0.722964741 | no |
| EPCAM | 1 | 1.31 | 7 | 0.88 | 0.520195839 | no |
| ERF | 0 | NA | 6 | 1.19 | 1 | no |
| ERG | 0 | 0 | 0 | 0 | 1 | no |
| ETV1 | 0 | 0 | 3 | 0.37 | 1 | no |
| FANCA | 0 | 0 | 33 | 4.05 | 0.064545683 | no |
| FGFR1 | 1 | 1.18 | 17 | 2.09 | 1 | no |
| FOXA1 | 0 | 0 | 0 | 0 | 1 | no |
| HOXB13 | 0 | 0 | 0 | 0 | 1 | no |
| IDH1 | 0 | 0 | 0 | NA | 1 | no |
| KMT2C | 1 | 1.37 | 8 | 0.99 | 0.54216491 | no |
| KMT2D | 0 | 0 | 1 | 0.12 | 1 | no |
| LYN | 0 | 0 | 2 | 0.38 | 1 | no |
| MED12 | 0 | 0 | 1 | 0.12 | 1 | no |
| MLH1 | 0 | 0 | 2 | 0.24 | 1 | no |
| MRE11A | 0 | 0 | 0 | 0 | 1 | no |
| MSH2 | 2 | 2.38 | 9 | 1.1 | 0.274981921 | no |
| MSH6 | 1 | 1.22 | 4 | 0.49 | 0.381852701 | no |
| MYC | 0 | 0 | 0 | 0 | 1 | no |
| NBN | 0 | 0 | 0 | 0 | 1 | no |
| NCOR1 | 0 | NA | 6 | 0.74 | 1 | no |
| PALB2 | 0 | 0 | 3 | 0.37 | 1 | no |
| PIK3CA | 0 | 0 | 0 | 0 | 1 | no |
| PMS2 | 0 | 0 | 0 | 0 | 1 | no |
| PRDM14 | 0 | 0 | 0 | 0 | 1 | no |
| PREX2 | 0 | 0 | 0 | 0 | 1 | no |
| PTEN | 12 | 14.12 | 169 | 20.76 | 0.15760421 | no |
| RAD21 | 0 | 0 | 0 | 0 | 1 | no |
| RAD50 | 1 | 1.25 | 2 | 0.25 | 0.246401253 | no |
| RAD51 | 0 | 0 | 2 | 0.24 | 1 | no |
| RAD51B | 0 | NA | 4 | 0.49 | 1 | no |
| RAD51C | 0 | 0 | 1 | 0.12 | 1 | no |
| RAD51D | 0 | 0 | 0 | 0 | 1 | no |
| RB1 | 15 | 17.65 | 42 | 5.16 | 0.000107505 | yes |
| RECQL4 | 0 | 0 | 1 | 0.13 | 1 | no |
| RNF43 | 1 | 1.23 | 3 | 0.37 | 0.317447599 | no |
| SPOP | 0 | 0 | 0 | 0 | 1 | no |
| TCEB1 | 0 | 0 | 0 | 0 | 1 | no |
| TMPRSS2 | 2 | 2.44 | 18 | 2.22 | 0.70517801 | no |
| TP53 | 4 | 4.71 | 31 | 3.8 | 0.564639213 | no |
| WHSC1L1 | 1 | 1.85 | 12 | 2.38 | 1 | no |
| ZFHX3 | 3 | 4.69 | 33 | 4.45 | 0.759187559 | no |

LOH

| **Gene** | **BRCA2^d^ (n)** | **Frequency**  **BRCA2^d^ (%)** | **BRCA2^i^ (n)** | **Frequency**  **BRCA2^i^ (%)** | **Nominal P-Value** | **Significant** |
| --- | --- | --- | --- | --- | --- | --- |
| AGO2 | 0 | 0 | 0 | 0 | 1 | no |
| AMER1 | 0 | 0 | 0 | 0 | 1 | no |
| APC | 1 | 1.18 | 1 | 0.12 | 0.180254725 | no |
| AR | 0 | 0 | 0 | 0 | 1 | no |
| ATM | 3 | 3.53 | 0 | 0 | 0.000818366 | yes |
| ATR | 0 | 0 | 0 | 0 | 1 | no |
| BARD1 | 0 | 0 | 0 | 0 | 1 | no |
| BRAF | 1 | 1.18 | 1 | 0.12 | 0.180254725 | no |
| BRCA1 | 1 | 1.18 | 0 | 0 | 0.094549499 | no |
| BRIP1 | 0 | 0 | 0 | 0 | 1 | no |
| CDK12 | 0 | 0 | 0 | 0 | 1 | no |
| CDKN1B | 2 | 2.44 | 2 | 0.25 | 0.043963042 | no |
| CHEK2 | 1 | 1.22 | 0 | 0 | 0.091517857 | no |
| CTNNB1 | 0 | 0 | 1 | 0.12 | 1 | no |
| CYSLTR2 | 0 | 0 | 0 | 0 | 1 | no |
| DUSP4 | 0 | 0 | 0 | 0 | 1 | no |
| EPCAM | 0 | 0 | 0 | 0 | 1 | no |
| ERF | 0 | NA | 0 | 0 | 1 | no |
| ERG | 0 | 0 | 0 | 0 | 1 | no |
| ETV1 | 1 | 1.23 | 0 | 0 | 0.090502793 | no |
| FANCA | 6 | 7.32 | 2 | 0.25 | 1.19E-05 | yes |
| FGFR1 | 2 | 2.35 | 1 | 0.12 | 0.024896089 | no |
| FOXA1 | 0 | 0 | 0 | 0 | 1 | no |
| HOXB13 | 0 | 0 | 0 | 0 | 1 | no |
| IDH1 | 1 | 1.18 | 0 | NA | 0.094549499 | no |
| KMT2C | 0 | 0 | 0 | 0 | 1 | no |
| KMT2D | 0 | 0 | 0 | 0 | 1 | no |
| LYN | 0 | 0 | 0 | 0 | 1 | no |
| MED12 | 1 | 1.24 | 0 | 0 | 0.090909091 | no |
| MLH1 | 0 | 0 | 1 | 0.12 | 1 | no |
| MRE11A | 2 | 2.47 | 0 | 0 | 0.008171604 | no |
| MSH2 | 0 | 0 | 1 | 0.12 | 1 | no |
| MSH6 | 0 | 0 | 1 | 0.12 | 1 | no |
| MYC | 0 | 0 | 0 | 0 | 1 | no |
| NBN | 0 | 0 | 0 | 0 | 1 | no |
| NCOR1 | 0 | NA | 0 | 0 | 1 | no |
| PALB2 | 0 | 0 | 1 | 0.12 | 1 | no |
| PIK3CA | 1 | 1.18 | 1 | 0.12 | 0.180254725 | no |
| PMS2 | 1 | 1.22 | 0 | 0 | 0.091517857 | no |
| PRDM14 | 0 | 0 | 0 | 0 | 1 | no |
| PREX2 | 0 | 0 | 0 | 0 | 1 | no |
| PTEN | 3 | 3.53 | 1 | 0.12 | 0.00304878 | yes |
| RAD21 | 0 | 0 | 0 | 0 | 1 | no |
| RAD50 | 1 | 1.25 | 0 | 0 | 0.08988764 | no |
| RAD51 | 2 | 2.47 | 0 | 0 | 0.008171604 | no |
| RAD51B | 0 | NA | 0 | 0 | 1 | no |
| RAD51C | 0 | 0 | 0 | 0 | 1 | no |
| RAD51D | 0 | 0 | 0 | 0 | 1 | no |
| RB1 | 8 | 9.41 | 3 | 0.37 | 6.09E-07 | yes |
| RECQL4 | 0 | 0 | 0 | 0 | 1 | no |
| RNF43 | 0 | 0 | 0 | 0 | 1 | no |
| SPOP | 0 | 0 | 0 | 0 | 1 | no |
| TCEB1 | 0 | 0 | 0 | 0 | 1 | no |
| TMPRSS2 | 3 | 3.66 | 0 | 0 | 0.000751201 | yes |
| TP53 | 3 | 3.53 | 2 | 0.25 | 0.007100907 | no |
| WHSC1L1 | 2 | 3.7 | 0 | 0 | 0.009175371 | no |
| ZFHX3 | 0 | 0 | 0 | 0 | 1 | no |

Gain

| **Gene** | **BRCA2^d^ (n)** | **Frequency**  **BRCA2^d^ (%)** | **BRCA2^i^ (n)** | **Frequency**  **BRCA2^i^ (%)** | **Nominal P-Value** | **Significant** |
| --- | --- | --- | --- | --- | --- | --- |
| AGO2 | 0 | 0 | 0 | 0 | 1 | no |
| AMER1 | 0 | 0 | 0 | 0 | 1 | no |
| APC | 0 | 0 | 1 | 0.12 | 1 | no |
| AR | 3 | 3.66 | 1 | 0.12 | 0.002767991 | yes |
| ATM | 0 | 0 | 0 | 0 | 1 | no |
| ATR | 2 | 2.47 | 0 | 0 | 0.008171604 | yes |
| BARD1 | 1 | 1.23 | 0 | 0 | 0.090909091 | no |
| BRAF | 1 | 1.18 | 1 | 0.12 | 0.180254725 | no |
| BRCA1 | 1 | 1.18 | 1 | 0.12 | 0.180254725 | no |
| BRIP1 | 6 | 7.32 | 1 | 0.12 | 3.21E-06 | yes |
| CDK12 | 1 | 1.23 | 0 | 0 | 0.090909091 | no |
| CDKN1B | 2 | 2.44 | 0 | 0 | 0.008282622 | yes |
| CHEK2 | 0 | 0 | 0 | 0 | 1 | no |
| CTNNB1 | 2 | 2.35 | 0 | 0 | 0.008844274 | yes |
| CYSLTR2 | 0 | 0 | 0 | 0 | 1 | no |
| DUSP4 | 0 | 0 | 0 | 0 | 1 | no |
| EPCAM | 1 | 1.31 | 0 | 0 | 0.087356322 | no |
| ERF | 0 | NA | 0 | 0 | 1 | no |
| ERG | 0 | 0 | 0 | 0 | 1 | no |
| ETV1 | 3 | 3.7 | 1 | 0.12 | 0.002677878 | yes |
| FANCA | 0 | 0 | 0 | 0 | 1 | no |
| FGFR1 | 1 | 1.18 | 2 | 0.25 | 0.257934043 | no |
| FOXA1 | 1 | 1.31 | 0 | 0 | 0.087356322 | no |
| HOXB13 | 5 | 6.49 | 0 | 0 | 5.95E-06 | yes |
| IDH1 | 1 | 1.18 | 0 | NA | 0.094549499 | no |
| KMT2C | 0 | 0 | 0 | 0 | 1 | no |
| KMT2D | 1 | 1.22 | 0 | 0 | 0.091517857 | no |
| LYN | 0 | 0 | 0 | 0 | 1 | no |
| MED12 | 1 | 1.24 | 0 | 0 | 0.090909091 | no |
| MLH1 | 2 | 2.35 | 0 | 0 | 0.008844274 | yes |
| MRE11A | 1 | 1.24 | 0 | 0 | 0.090909091 | no |
| MSH2 | 1 | 1.19 | 0 | 0 | 0.093541203 | no |
| MSH6 | 1 | 1.22 | 0 | 0 | 0.091517857 | no |
| MYC | 8 | 9.76 | 2 | 0.25 | 1.38E-07 | yes |
| NBN | 7 | 8.54 | 2 | 0.25 | 1.31E-06 | yes |
| NCOR1 | 0 | NA | 0 | 0 | 1 | no |
| PALB2 | 1 | 1.19 | 0 | 0 | 0.093541203 | no |
| PIK3CA | 5 | 5.88 | 0 | 0 | 6.78E-06 | yes |
| PMS2 | 3 | 3.66 | 1 | 0.12 | 0.002767991 | yes |
| PRDM14 | 0 | 0 | 0 | 0 | 1 | no |
| PREX2 | 0 | 0 | 0 | 0 | 1 | no |
| PTEN | 0 | 0 | 0 | 0 | 1 | no |
| RAD21 | 8 | 10.67 | 2 | 0.27 | 1.28E-07 | yes |
| RAD50 | 0 | 0 | 0 | 0 | 1 | no |
| RAD51 | 1 | 1.23 | 0 | 0 | 0.090909091 | no |
| RAD51B | 0 | NA | 0 | 0 | 1 | no |
| RAD51C | 4 | 4.82 | 0 | 0 | 6.98E-05 | yes |
| RAD51D | 1 | 1.23 | 0 | 0 | 0.090909091 | no |
| RB1 | 0 | 0 | 0 | 0 | 1 | no |
| RECQL4 | 8 | 10.39 | 0 | 0 | 2.65E-09 | yes |
| RNF43 | 4 | 4.94 | 0 | 0 | 6.38E-05 | yes |
| SPOP | 4 | 4.94 | 0 | 0 | 6.38E-05 | yes |
| TCEB1 | 6 | 8.33 | 0 | 0 | 3.94E-07 | yes |
| TMPRSS2 | 0 | 0 | 0 | 0 | 1 | no |
| TP53 | 0 | 0 | 0 | 0 | 1 | no |
| WHSC1L1 | 1 | 1.85 | 0 | 0 | 0.096601073 | no |
| ZFHX3 | 0 | 0 | 0 | 0 | 1 | no |

Amplification

| **Gene** | **BRCA2^d^ (n)** | **Frequency**  **BRCA2^d^ (%)** | **BRCA2^i^ (n)** | **Frequency**  **BRCA2^i^ (%)** | **Nominal P-Value** | **Significant** |
| --- | --- | --- | --- | --- | --- | --- |
| AGO2 | 5 | 11.11 | 29 | 5.74 | 0.184654687 | no |
| AMER1 | 3 | 4.11 | 54 | 6.66 | 0.616428627 | no |
| APC | 0 | 0 | 0 | 0 | 1 | no |
| AR | 12 | 14.63 | 236 | 28.99 | 0.004366385 | no |
| ATM | 1 | 1.18 | 0 | 0 | 0.094549499 | no |
| ATR | 2 | 2.47 | 6 | 0.74 | 0.159693923 | no |
| BARD1 | 0 | 0 | 0 | 0 | 1 | no |
| BRAF | 1 | 1.18 | 4 | 0.49 | 0.392120726 | no |
| BRCA1 | 0 | 0 | 2 | 0.24 | 1 | no |
| BRIP1 | 0 | 0 | 6 | 0.74 | 1 | no |
| CDK12 | 0 | 0 | 1 | 0.12 | 1 | no |
| CDKN1B | 0 | 0 | 1 | 0.12 | 1 | no |
| CHEK2 | 0 | 0 | 0 | 0 | 1 | no |
| CTNNB1 | 0 | 0 | 2 | 0.25 | 1 | no |
| CYSLTR2 | 0 | 0 | 0 | 0 | 1 | no |
| DUSP4 | 0 | 0 | 0 | 0 | 1 | no |
| EPCAM | 0 | 0 | 1 | 0.13 | 1 | no |
| ERF | 0 | NA | 0 | 0 | 1 | no |
| ERG | 1 | 1.22 | 2 | 0.25 | 0.251464613 | no |
| ETV1 | 2 | 2.47 | 5 | 0.62 | 0.126059766 | no |
| FANCA | 0 | 0 | 0 | 0 | 1 | no |
| FGFR1 | 2 | 2.35 | 20 | 2.46 | 1 | no |
| FOXA1 | 3 | 3.94 | 13 | 1.64 | 0.157346151 | no |
| HOXB13 | 0 | 0 | 8 | 1.06 | 1 | no |
| IDH1 | 0 | 0 | 0 | NA | 1 | no |
| KMT2C | 0 | 0 | 2 | 0.25 | 1 | no |
| KMT2D | 0 | 0 | 0 | 0 | 1 | no |
| LYN | 3 | 6 | 20 | 3.85 | 0.443509535 | no |
| MED12 | 0 | 0 | 18 | 2.23 | 0.39670287 | no |
| MLH1 | 0 | 0 | 2 | 0.24 | 1 | no |
| MRE11A | 1 | 1.24 | 1 | 0.12 | 0.173646578 | no |
| MSH2 | 0 | 0 | 1 | 0.12 | 1 | no |
| MSH6 | 0 | 0 | 1 | 0.12 | 1 | no |
| MYC | 14 | 17.07 | 83 | 10.19 | 0.062682658 | no |
| NBN | 7 | 8.54 | 46 | 5.65 | 0.320578008 | no |
| NCOR1 | 0 | NA | 3 | 0.37 | 1 | no |
| PALB2 | 0 | 0 | 2 | 0.25 | 1 | no |
| PIK3CA | 0 | 0 | 1 | 0.12 | 1 | no |
| PMS2 | 2 | 2.44 | 3 | 0.37 | 0.068963328 | no |
| PRDM14 | 5 | 11.11 | 40 | 7.92 | 0.400538946 | no |
| PREX2 | 5 | 10 | 36 | 6.92 | 0.390989957 | no |
| PTEN | 0 | 0 | 0 | 0 | 1 | no |
| RAD21 | 7 | 9.33 | 36 | 4.8 | 0.100970359 | no |
| RAD50 | 0 | 0 | 0 | 0 | 1 | no |
| RAD51 | 0 | 0 | 2 | 0.24 | 1 | no |
| RAD51B | 0 | NA | 0 | 0 | 1 | no |
| RAD51C | 0 | 0 | 4 | 0.5 | 1 | no |
| RAD51D | 0 | 0 | 1 | 0.12 | 1 | no |
| RB1 | 0 | 0 | 0 | 0 | 1 | no |
| RECQL4 | 9 | 11.69 | 36 | 4.53 | 0.013146678 | no |
| RNF43 | 0 | 0 | 3 | 0.37 | 1 | no |
| SPOP | 0 | 0 | 1 | 0.12 | 1 | no |
| TCEB1 | 10 | 13.89 | 46 | 6.2 | 0.024313829 | no |
| TMPRSS2 | 0 | 0 | 2 | 0.25 | 1 | no |
| TP53 | 0 | 0 | 0 | 0 | 1 | no |
| WHSC1L1 | 2 | 3.7 | 9 | 1.78 | 0.288020323 | no |
| ZFHX3 | 0 | 0 | 0 | 0 | 1 | no |

**Supplementary Table 13**. CNA frequency in primary versus metastatic *BRCA2^d^* tumors

Homozygous Deletion

| **Gene** | **Primary (n)** | **Frequency**  **Primary (%)** | **Metastatic (n)** | **Frequency**  **Metastatic (%)** | **Nominal P-Value** | **Significant** |
| --- | --- | --- | --- | --- | --- | --- |
| APC | 0 | 0 | 4 | 4.94 | 0.127703 | no |
| AR | 0 | 0 | 0 | 0 | 1 | no |
| ATM | 0 | 0 | 2 | 2.47 | 0.500453 | no |
| ATR | 0 | 0 | 0 | 0 | 1 | no |
| BRAF | 0 | 0 | 0 | 0 | 1 | no |
| CDKN1B | 2 | 2.94 | 2 | 2.56 | 1 | no |
| EPCAM | 1 | 1.82 | 1 | 1.39 | 1 | no |
| ERG | 0 | 0 | 0 | 0 | 1 | no |
| ETV1 | 0 | 0 | 0 | 0 | 1 | no |
| FANCA | 3 | 4.41 | 0 | 0 | 0.098638 | no |
| FOXA1 | 0 | 0 | 0 | 0 | 1 | no |
| MRE11A | 0 | 0 | 0 | 0 | 1 | no |
| MSH2 | 1 | 1.47 | 1 | 1.25 | 1 | no |
| MSH6 | 0 | 0 | 1 | 1.28 | 1 | no |
| MYC | 0 | 0 | 0 | 0 | 1 | no |
| NBN | 0 | 0 | 0 | 0 | 1 | no |
| PIK3CA | 0 | 0 | 0 | 0 | 1 | no |
| PMS2 | 0 | 0 | 0 | 0 | 1 | no |
| PTEN | 1 | 1.47 | 10 | 12.35 | 0.011958 | no |
| RAD50 | 0 | 0 | 1 | 1.32 | 1 | no |
| RB1 | 3 | 4.41 | 13 | 16.05 | 0.031684 | no |
| RNF43 | 0 | 0 | 1 | 1.3 | 1 | no |
| TMPRSS2 | 0 | 0 | 2 | 2.56 | 0.502128 | no |
| TP53 | 0 | 0 | 4 | 4.94 | 0.125666 | no |

Amplification

| **Gene** | **Primary (n)** | **Frequency**  **Primary (%)** | **Metastatic (n)** | **Frequency**  **Metastatic (%)** | **Nominal P-Value** | **Significant** |
| --- | --- | --- | --- | --- | --- | --- |
| APC | 0 | 0 | 0 | 0 | 1 | no |
| AR | 3 | 4.41 | 12 | 15.38 | 0.03216 | no |
| ATM | 0 | 0 | 1 | 1.23 | 1 | no |
| ATR | 0 | 0 | 2 | 2.6 | 0.504079 | no |
| BRAF | 0 | 0 | 1 | 1.23 | 1 | no |
| CDKN1B | 0 | 0 | 0 | 0 | 1 | no |
| EPCAM | 0 | 0 | 0 | 0 | 1 | no |
| ERG | 0 | 0 | 1 | 1.28 | 1 | no |
| ETV1 | 1 | 1.52 | 2 | 2.6 | 1 | no |
| FANCA | 0 | 0 | 0 | 0 | 1 | no |
| FOXA1 | 1 | 1.82 | 3 | 4.17 | 0.632724 | no |
| MRE11A | 0 | 0 | 1 | 1.3 | 1 | no |
| MSH2 | 0 | 0 | 0 | 0 | 1 | no |
| MSH6 | 0 | 0 | 0 | 0 | 1 | no |
| MYC | 5 | 7.35 | 14 | 17.95 | 0.083171 | no |
| NBN | 5 | 7.35 | 7 | 8.97 | 0.771595 | no |
| PIK3CA | 1 | 1.47 | 0 | 0 | 0.456376 | no |
| PMS2 | 0 | 0 | 2 | 2.56 | 0.498914 | no |
| PTEN | 0 | 0 | 0 | 0 | 1 | no |
| RAD50 | 0 | 0 | 0 | 0 | 1 | no |
| RB1 | 0 | 0 | 0 | 0 | 1 | no |
| RNF43 | 0 | 0 | 0 | 0 | 1 | no |
| TMPRSS2 | 0 | 0 | 0 | 0 | 1 | no |
| TP53 | 0 | 0 | 0 | 0 | 1 | no |

**Supplementary Table 14.** *TMPRSS2* and/or ETS-related structural variants in TCGA and GENIE. Table presents number of samples with given fusion among primary and metastatic *BRCA2^d^* and *BRCA2^i^*. Inferred SV is determined based on gene proximity (<2Mbp) to *TMPRSS2* or ETS family gene.

| **Original SV** | **Inferred SV** | **Total n** | **Primary *BRCA2^d^* n** | **Primary *BRCA2^i^* n** | **Metastatic *BRCA2^d^* n** | **Metastatic *BRCA2^i^* n** |
| --- | --- | --- | --- | --- | --- | --- |
| ELF3-intragenic | ELF3-intragenic | 1 | 0 | 0 | 0 | 1 |
| ELK4-SLC26A9 | ELK4-intragenic | 1 | 0 | 1 | 0 | 0 |
| ERG-CLDN14 | ERG-intragenic | 1 | 0 | 0 | 0 | 1 |
| ERG-intragenic | ERG-intragenic | 1 | 0 | 1 | 0 | 0 |
| ERG-KCNJ6 | ERG-intragenic | 1 | 0 | 1 | 0 | 0 |
| ERG-SLC45A3 | ERG-SLC45A3 | 12 | 0 | 12 | 0 | 0 |
| ETV1-intragenic | ETV1-intragenic | 1 | 0 | 1 | 0 | 0 |
| ETV1-KLK2 | ETV1-SPIB | 1 | 0 | 1 | 0 | 0 |
| ETV1-SLC45A3 | ETV1-SLC45A3 | 7 | 0 | 7 | 0 | 0 |
| ETV4-STAT3 | ETV4-intragenic | 1 | 0 | 1 | 0 | 0 |
| ETV6-BCL2L14 | ETV6-intragenic | 1 | 0 | 0 | 0 | 1 |
| ETV6-EMP1 | ETV6-intragenic | 1 | 0 | 1 | 0 | 0 |
| ETV6-intragenic | ETV6-intragenic | 2 | 0 | 2 | 0 | 0 |
| TMPRSS2-DGKG | TMPRSS2-ETV5 | 2 | 0 | 2 | 0 | 0 |
| TMPRSS2-DSCAM | TMPRSS2-intragenic | 2 | 0 | 0 | 0 | 2 |
| TMPRSS2-DYRK1A | TMPRSS2-ETS2 | 1 | 0 | 1 | 0 | 0 |
| TMPRSS2-ERG | TMPRSS2-ERG | 746 | 19 | 503 | 15 | 209 |
| TMPRSS2-ETV1 | TMPRSS2-ETV1 | 10 | 3 | 4 | 2 | 1 |
| TMPRSS2-ETV4 | TMPRSS2-ETV4 | 11 | 0 | 8 | 0 | 3 |
| TMPRSS2-ETV5 | TMPRSS2-ETV5 | 4 | 0 | 3 | 0 | 1 |
| TMPRSS2-intragenic | TMPRSS2-intragenic | 53 | 1 | 30 | 1 | 21 |
| TMPRSS2-KCNJ6 | TMPRSS2-ERG | 1 | 0 | 1 | 0 | 0 |
| TMPRSS2-LINC00114 | TMPRSS2-ERG | 12 | 0 | 9 | 0 | 3 |
| TMPRSS2-PLCD3 | TMPRSS2-ETV4 | 2 | 0 | 1 | 0 | 1 |
| TMPRSS2-PRDM15 | TMPRSS2-intragenic | 1 | 1 | 0 | 0 | 0 |
| TMPRSS2-SH3BGR | TMPRSS2-ETS2 | 1 | 0 | 0 | 0 | 1 |
| TMPRSS2-SIK1 | TMPRSS2-intragenic | 1 | 0 | 1 | 0 | 0 |
| TMPRSS2-SLC45A3 | TMPRSS2-ELK4 | 2 | 0 | 1 | 0 | 1 |
| TMPRSS2-U2AF1 | TMPRSS2-intragenic | 1 | 0 | 1 | 0 | 0 |
