# Supplementary material for: The tumor mutational landscape of BRCA2-deficient primary and metastatic prostate cancer
Source: NPJ Precis Oncol. 2022 Jun 17;6:39. doi: 10.1038/s41698-022-00284-6 (PMC9205939; doi:10.1038/s41698-022-00284-6)
Supplement: Supplementary file 2 — REPORTING SUMMARY [file 41698_2022_284_MOESM2_ESM.pdf]

nature portfolio

Corresponding author(s): TR Rebbeck, NPJPRECISIONONCOLOGY-01727R

Last updated by author(s): Feb 17, 2022

Reporting Summary

Nature Portfolio wishes to improve the reproducibility of the work that we publish. This form provides structure for consistency and transparency in reporting. For further information on Nature Portfolio policies, see our Editorial Policies and the Editorial Policy Checklist.

Statistics

For all statistical analyses, confirm that the following items are present in the figure legend, table legend, main text, or Methods section.

n/a	Confirmed	
☐	☒	The exact sample size (*n*) for each experimental group/condition, given as a discrete number and unit of measurement
☐	☒	A statement on whether measurements were taken from distinct samples or whether the same sample was measured repeatedly
☐	☒	The statistical test(s) used AND whether they are one- or two-sided<br>*Only common tests should be described solely by name; describe more complex techniques in the Methods section.*
☐	☒	A description of all covariates tested
☐	☒	A description of any assumptions or corrections, such as tests of normality and adjustment for multiple comparisons
☐	☒	A full description of the statistical parameters including central tendency (e.g. means) or other basic estimates (e.g. regression coefficient) AND variation (e.g. standard deviation) or associated estimates of uncertainty (e.g. confidence intervals)
☐	☒	For null hypothesis testing, the test statistic (e.g. *F*, *t*, *r*) with confidence intervals, effect sizes, degrees of freedom and *P* value noted<br>*Give P values as exact values whenever suitable.*
☒	☐	For Bayesian analysis, information on the choice of priors and Markov chain Monte Carlo settings
☒	☐	For hierarchical and complex designs, identification of the appropriate level for tests and full reporting of outcomes
☒	☐	Estimates of effect sizes (e.g. Cohen's *d*, Pearson's *r*), indicating how they were calculated

Our web collection on statistics for biologists contains articles on many of the points above.

Software and code

Policy information about availability of computer code

Data collection	No data collection was performed in this study. All data were obtained from publicly-available databases.
Data analysis	Data analysis was conducted using R (version 4.0.5) and Python (version 3.8.5). Maftools (version 2.8.05), sigminer (version 2.1.1), and custom scripts were used to complete the analyses.

For manuscripts utilizing custom algorithms or software that are central to the research but not yet described in published literature, software must be made available to editors and reviewers. We strongly encourage code deposition in a community repository (e.g. GitHub). See the Nature Portfolio guidelines for submitting code & software for further information.

Data

Policy information about availability of data

All manuscripts must include a data availability statement. This statement should provide the following information, where applicable:

- Accession codes, unique identifiers, or web links for publicly available datasets
- A description of any restrictions on data availability
- For clinical datasets or third party data, please ensure that the statement adheres to our policy

The data that support the findings of this study are available from the AACR Project GENIE (https://www.synapse.org/#!Synapse:syn7222066/wiki/405659), the International Cancer Genome Consortium (https://dcc.icgc.org/releases/release_28), and The Cancer Genome Atlas (https://www.cbioportal.org/study/summary?id=prad_tcga_pan_can_atlas_2018).

Field-specific reporting

Please select the one below that is the best fit for your research. If you are not sure, read the appropriate sections before making your selection.

☒ Life sciences ☐ Behavioural & social sciences ☐ Ecological, evolutionary & environmental sciences

For a reference copy of the document with all sections, see nature.com/documents/nr-reporting-summary-flat.pdf

Life sciences study design

All studies must disclose on these points even when the disclosure is negative.

Sample size	Sample size was limited by data availability in the ICGC, GENIE, and TCGA databases. This represents the single largest dataset to characterize the BRCA2-deficient prostate cancer phenotype.
Data exclusions	Data were excluded from non-tumor samples, replicate samples from the same patient, samples that did not include profiling for BRCA2, and samples without appropriate profiling of the genomic alteration types of interest.
Replication	To our knowledge, there are no other large publicly available datasets in which these analyses could be replicated. Given the frequency of BRCA2-deficiency, it was necessary to pool the ICGC, GENIE, and TCGA to obtain a sufficient large sample of BRCA2-deficient prostate tumors to characterize the phenotype.
Randomization	No randomization was performed. This was an observational study examining differences in the tumor mutational landscape between BRCA2-deficient and BRCA2-intact prostate tumors.
Blinding	The authors did not collect the data and were not blinded to tumor BRCA2 status during the analyses.

Reporting for specific materials, systems and methods

We require information from authors about some types of materials, experimental systems and methods used in many studies. Here, indicate whether each material, system or method listed is relevant to your study. If you are not sure if a list item applies to your research, read the appropriate section before selecting a response.

Materials & experimental systems

n/a	Involved in the study	
☒	☐	Antibodies
☒	☐	Eukaryotic cell lines
☒	☐	Palaeontology and archaeology
☒	☐	Animals and other organisms
☐	☒	Human research participants
☒	☐	Clinical data
☒	☐	Dual use research of concern

Methods

n/a	Involved in the study	
☒	☐	ChIP-seq
☒	☐	Flow cytometry
☒	☐	MRI-based neuroimaging

Human research participants

Policy information about studies involving human research participants

Population characteristics	Data included in this study was from adult males with primary and/or metastatic prostate cancer with tumor genomic data deposited in the AACR Project GENIE, the International Cancer Genome Consortium, and The Cancer Genome Atlas databases
Recruitment	Recruitment of participants was conducted by the AACR Project GENIE, the International Cancer Genome Consortium, and The Cancer Genome Atlas Research Network. These are not population-based datasets and may have limited generalizability.
Ethics oversight	This study utilized de-identified data from the AACR Project GENIE, the International Cancer Genome Consortium, and The Cancer Genome Atlas.

Note that full information on the approval of the study protocol must also be provided in the manuscript.
